# Development and validation of a decision model for the evaluation of novel lung cancer treatments in the Netherlands

**DOI:** 10.1038/s41598-023-29286-5

**Published:** 2023-02-09

**Authors:** Zakile A. Mfumbilwa, Janneke A. Wilschut, Martijn J. H. G. Simons, Bram Ramaekers, Manuela Joore, Valesca Retèl, Christine M. Cramer-van der Welle, Franz M. N. H. Schramel, Ewoudt M. W. van de Garde, Veerle M. H. Coupé

**Affiliations:** 1 Department of Epidemiology and Data Science, Amsterdam Public Health, Amsterdam UMC, Vrije Universiteit Amsterdam, de Boelelaan 1117, PO Box 7057, 1007 MB Amsterdam, The Netherlands; 2 Department of Clinical Epidemiology and Medical Technology Assessment, Maastricht University Medical Centre+, Maastricht, The Netherlands; 3 Maastricht University, Care And Public Health Research Institute (CAPHRI), Maastricht, The Netherlands; 4 Department of Health Technology and Services Research, University of Twente, Enschede, The Netherlands; 5 Santeon Hospital Group, Santeon, Herculesplein 38, 3584 AA Utrecht, The Netherlands; 6 Division of Pharmacoepidemiology and Clinical Pharmacology, Department of Pharmaceutical Sciences, Utrecht University, Utrecht, The Netherlands; 7 Department of Clinical Pharmacy, St. Antonius Hospital, Utrecht, Nieuwegein, The Netherlands

**Keywords:** Non-small-cell lung cancer, Health care economics, Non-small-cell lung cancer, Outcomes research

## Abstract

Recent discoveries in molecular diagnostics and drug treatments have improved the treatment of patients with advanced (inoperable) non-squamous non-small cell lung cancer (NSCLC) from solely platinum-based chemotherapy to more personalized treatment, including targeted therapies and immunotherapies. However, these improvements come at considerable costs, highlighting the need to assess their cost-effectiveness in order to optimize lung cancer care. Traditionally, cost-effectiveness models for the evaluation of new lung cancer treatments were based on the findings of the randomized control trials (RCTs). However, the strict RCT inclusion criteria make RCT patients not representative of patients in the real-world. Patients in RCTs have a better prognosis than patients in a real-world setting. Therefore, in this study, we developed and validated a diagnosis-treatment decision model for patients with advanced (inoperable) non-squamous NSCLC based on real-world data in the Netherlands. The model is a patient-level microsimulation model implemented as discrete event simulation with five health events. Patients are simulated from diagnosis to death, including at most three treatment lines. The base-model (non-personalized strategy) was populated using real-world data of patients treated with platinum-based chemotherapy between 2008 and 2014 in one of six Dutch teaching hospitals. To simulate personalized care, molecular tumor characteristics were incorporated in the model based on the literature. The impact of novel targeted treatments and immunotherapies was included based on published RCTs. To validate the model, we compared survival under a personalized treatment strategy with observed real-world survival. This model can be used for health-care evaluation of personalized treatment for patients with advanced (inoperable) NSCLC in the Netherlands.

## Introduction

The treatment of patients with advanced (inoperable) non-small cell lung cancer (NSCLC) has changed drastically in the last decade. New medicines have broadened the options for first-line treatment from solely platinum-based chemotherapy to also targeted therapies and immunotherapies^1^. Likewise, the diagnostic pathway has changed to aid patient selection for optimal treatment decision making, which has resulted in a more personalized treatment scheme^1,2^.

These innovations were supported by increased understanding of the biology and molecular subtypes of NSCLC. As a consequence, NSCLC has been defined as a heterogeneous disease consisting of molecularly defined tumor subgroups that require personalized biomarker-guided treatment selection^3,4^. For some NSCLC tumor subgroups, targeted treatment and immunotherapies have been shown to improve progression-free survival (PFS) and/or overall survival (OS)^4^.

Although there are improvements in survival, they are accompanied by a substantial increase in costs for both molecular diagnostics and drugs^5^. To support treatment decisions that optimize budget allocation so that health benefits are maximized, decision models are commonly recommended^6,7^. Decision models simplify complex systems and allow integration of data from different sources as well as extrapolation of short-term effects to long-term outcomes^6–8^. Decision models are commonly used for cost-effectiveness analyses and budget impact analyses to evaluate diagnostic and treatment decisions in different scenarios^9^.

For personalized treatment of advanced lung cancer care, cost-effectiveness and budget impact evaluations have been carried out based on randomized controlled trials (RCTs)^10–15^. Holleman et al.^10^, Chouaid et al.^15^, Barbier et al.^11^, and Westwood et al.^12^ have taken a customary approach of modelling single drug(s) for a specific indication or a drug-diagnostics combination. To optimize the whole care pathway, the diagnostic and treatment pathways must be evaluated over multiple treatment lines. Thus, RCTs on single interventions are no longer sufficient. Simons et al.^13^ and van Amerongen et al.^14^ modelled the diagnostic and treatment pathways in multiple lines based on data from several RCTs. However, RCTs represent highly selective populations that generally have a better prognosis than patients not participating in RCTs^16–18^. It has been shown , for example, that the real-world overall survival (OS) of patients with high programmed death ligand 1 (PD-L1) expression who received first-line immunotherapy treatment is shorter than the OS in RCTs^17,19–21^. It is therefore unclear whether the predictions of decision models simulating novel diagnostics and treatments based on the RCT setting translate well to the real-world clinical setting. To understand this, it is important to build decision models based on real-world data.

In the Netherlands, decision models based on real-world data have been developed for other tumor types. For example, an economic evaluation for advanced breast cancer treatment has been performed based on the Southeast Netherlands advanced breast cancer registry^22^. The study presented here aims to develop and validate a lung cancer diagnosis and treatment decision model for patients with advanced (inoperable) non-squamous NSCLC based on real-world data in the Netherlands that can be used for economic evaluations of diagnostics and personalized treatment in the Netherlands.

## Materials and methods

### Overview of the microsimulation model

**Figure 1:**
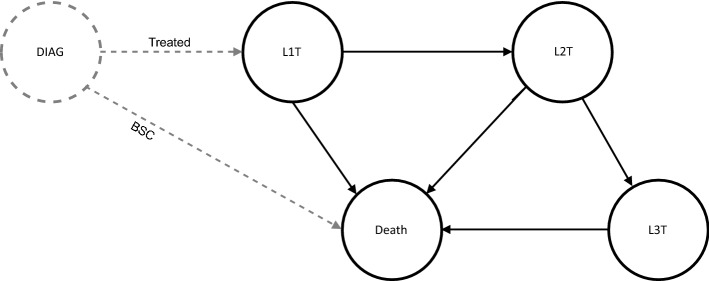
Microsimulation diagnosis-treatment model. DIAG, diagnosis; L1T, L2T, and L3T are start of first-, second-, and third-line treatment, respectively; BSC, best supportive care, i.e., patients who are ineligible for L1T; Treated, patients who started at least one line of systemic treatment. Arrows indicate the possible transitions from one event to the start of a next treatment line or death. Black circles and lines denote the disease trajectory after treatment initiation. The corresponding time to event distributions were modelled as a parametric multistate statistical model (parMSSM). Gray dotted circle and lines denote the trajectory from DIAG to either start of treatment or death. Time-to-events from DIAG to L1T and from DIAG to death were not part of parMSSM.

We developed a patient-level microsimulation model implemented as a discrete event simulation (DES). Patients with advanced (inoperable) non-squamous NSCLC are simulated from diagnosis to death, including at most three treatment lines (Fig. 1). We adopted a patient-level simulation framework to capture the complexity of the disease by modelling the patients’ characteristics (attributes) and their treatment history leading to outcome variation on a patient level^23^. In addition, DES allows time to events to vary by patient by directly sampling event times from parametric distributions^24^.

The model incorporates three life-prolonging treatment lines and five health events:

- DIAG, diagnosis of advanced (inoperable) non-squamous NSCLC.
- L1T, start of first-line systemic treatment.
- L2T, start of second-line systemic treatment.
- L3T, start of third-line systemic treatment.
- Death, death (absorption event).

All patients start at DIAG and reach the next event in a continuous-time framework. From DIAG to L3T, a patient can start a subsequent treatment line or die. Not every patient passes all events; a patient may die before reaching the subsequent treatment line. A patient may die from the disease or from death due to other causes (DoC).

**Figure 2:**
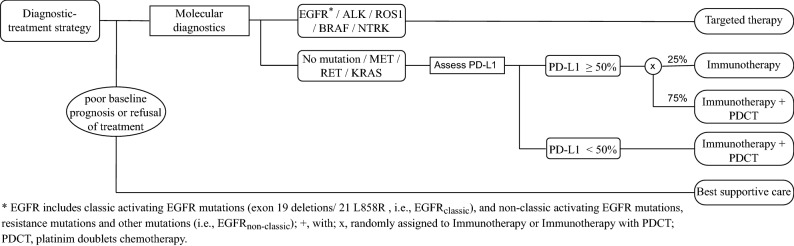
Molecular diagnosis-treatment decision tree.

The model was developed in two steps. First, we developed a model simulating a non-personalized treatment strategy that reflects a real-world disease trajectory under either standard systemic chemotherapy regimens commonly used before 2014 or best supportive care (BSC). Second, the model parameters were adjusted to simulate a personalized treatment strategy. Adjustment includes adding a molecular diagnosis-treatment decision tree (Fig. 2). The molecular diagnosis-treatment decision tree simulates the distribution of molecular biomarkers that are used to inform first-line treatment choice. In the personalized treatment strategy, patients can receive targeted therapies and immunotherapies, dependent on the presence of these molecular biomarkers.

Below, the data used for model quantification, model development and internal and external validation are described in detail.

### Data used for parameter estimation

#### Santeon registry 2008–2014 (model development based on a non-personalized treatment strategy)

Data from six Dutch teaching hospitals working under the Santeon group^25^ were used. This dataset includes patients with advanced (inoperable) NSCLC diagnosed and treated between 2008 and 2014 (Santeon registry 2008 – 2014). Patients were followed until January 31, 2017^18^. Between 2008 and 2014, platinum-based chemotherapies were the standard of care. At that time, first-line immunotherapy was not among the treatment options, while targeted therapy was introduced in the last phase of 2008–2014^26–29^.

For model development, we used the subset of 2196 patients with non-squamous histology. The median (range) follow-up time was 59 months (0–106). At the end-date of follow-up, 98 percent of the 2196 patients had died.

We used the following patient characteristics as model attributes: year of diagnosis (year), age at diagnosis (age), sex, Eastern Cooperative Oncology Group (ECOG) performance status (PS), and Charlson comorbidity index (CCI). Patients received either best supportive care (BSC) or one or more lines of systemic chemotherapy. Patients were excluded if they had squamous cell carcinoma (527), were treated with targeted or unspecified therapy in any of the treatment lines (258), or had inconsistency in the event times (1). The descriptive statistics of the 2196 patients and the bivariate associations among covariates are given in electronic supplementary materials (ESM) Tables A1 and A2. Throughout this manuscript, we will refer to this dataset as the *“template dataset”.*

#### Molecular characteristics and effects of novel treatment

The prevalence of molecular biomarkers used to aid personalized treatment decisions was taken from the scientific literature^30–33^ (see ESM Table A3). The molecular biomarkers included activating mutation of the epidermal growth factor receptor (EGFR) gene, anaplastic lymphoma kinase (ALK) gene rearrangements, genetic aberrations of ROS proto-oncogene 1 (ROS1), B-Raf proto-oncogene (BRAF), MET proto-oncogene (MET), RET proto-oncogene (RET), neurotrophic receptor tyrosine kinase 1 (NTRK_(1, 2, 3)_) and Kirsten rat sarcoma viral oncogene homolog (KRAS). Furthermore, PD-L1 protein expression^3^ was included. With respect to EGFR mutations, a distinction was made between classic activating EGFR mutations (exon 19 deletions and exon 21 L858R point mutations, i.e., EGFR_classic_) and non-classic activating EGFR mutations, resistance mutations, and other mutations (EGFR_non-classic_)^31^.

The impact of novel treatments in terms of reducing the hazard rate to progression compared to standard chemotherapy per molecular subgroup and per treatment line was taken from randomized controlled trials (RCTs) (i.e., direct treatment comparisons^34–38^, a network meta-analysis (NMA)^39^ and a systematic review providing a pooled estimate^40^). See ESM Tables A4 and A5.

#### Validation datasets

The strategy that simulates personalized treatment was externally validated using PFS and OS curves for different therapies from the Santeon registry 2015–2018^19^. Data from Cramer-van der Welle et al.^19^ were reconstructed using the method described by Hoyle & Henley^41^. The reconstructed data were 1) the OS of 147 patients with an EGFR_classic_ mutation treated with first-line EGFR tyrosine kinase inhibitor (EGFR-TKI, i.e., gefitinib or erlotinib). 2) the PFS and the OS of 83 patients with PD-L1 ≥ 50% treated with first-line pembrolizumab monotherapy. Patient characteristics are provided in Cramer-van der Welle et al.^19^. Additionally, we used patient-level data of 52 patients with known EGFR_classic_ mutations treated with first-line EGFR-TKIs (gefitinib or erlotinib) in the Santeon registry 2008–2014^42^. Their characteristics are given in Slug et al.^42^. Lastly, published data from the United States (US)^17,21^ were used to validate model projections for the subgroup of patients treated with a combination of pembrolizumab and chemotherapy for patients with PD-L1 ≥ 50% and PD-L1 1–49%.

### Parameterization of the non-personalized strategy

Parameters for the non-personalized strategy were directly estimated from *the template dataset.* We used the chi-square test, t-test, and one-way ANOVA to assess the bivariate association between baseline patient characteristics and treatment decisions after diagnosis. Logistic and linear regression models were used to sample a treatment decision based on the influential patient characteristics (ESM Table A6).

Parameters for sampling a patients’ time to event were estimated by fitting parametric survival models. First, from DIAG to L1T and to Death *(Fig. *1*, dotted circle and lines*), two independent parametric distributions were used to describe time to L1T for the patients receiving life-prolonging systemic treatment (first-line treatment) and time to death for the BSC group as a function of baseline attributes. This means that first-line treatment and BSC were not considered competing events. The following survival functions were considered: exponential, Weibull, Gompertz, log-logistics, log-normal, and generalized gamma. Based on visual inspection^43,44^, log-logistic and log-normal distributions had the best fit from DIAG to L1T and DIAG to death, respectively.

Second, after the start of first-line treatment (from L1T to L2T and to death; from L2T to L3T and to death; and from L3T to death, *Fig. *1*, solid circles and lines*), the patients’ disease trajectory was estimated by a *parametric multistate statistical model* (parMSSM) adjusted for patients’ baseline attributes^45,46^. In this study, we evaluated three proportional hazard (PH) distribution functions (Exponential, Weibull, and Gompertz). The optimal distribution was chosen based on visual inspection. The Gompertz distribution had the best fit for time from L1T to L2T and L1T to death, while the exponential distribution was optimal for time from L2T to L3T and L2T to death. Because of the limited number of patients, the exponential distribution was selected for the time from L3T to death. The patient attributes influencing the time to event were selected based on backward selection (cutoff p-value < 0.05).

Time to death was corrected for background mortality (DoC), described by age- and sex-specific life tables for the general Dutch population adjusted for smoking^47^.

Data analysis and the construction of the microsimulation model were performed using statistical software R, version 4.0.2^48^. For time-to-event data analysis, the dataset was managed using the “msprep” function of the mstate package^49^ and analyzed using the “phreg” function of the eha package^50^ and the “flexreg” function of the flexsurv package^51^.

For details on parameter estimation and microsimulation model development, see ESM.

### Adjustment of the non-personalized strategy to simulate a personalized treatment strategy

To simulate a personalized treatment strategy, we adjusted the non-personalized strategy by adding information on molecular biomarkers currently used to aid treatment decisions, as shown in Fig. 2. In the model, we assumed that molecular biomarkers are independent of clinical and pathological characteristics. The molecular biomarkers included are described in Sect. “Molecular characteristics and effects of novel treatment”, and their prevalence is given in ESM Table A3.

Subsequently, first-line systemic treatment as well as second-line treatment was adapted to project outcomes under the personalized strategy according to the decision tree shown in Fig. 2^1^. The decision tree can flexibly be specified in the model, detailing the type of treatment as a function of the presence of molecular biomarkers in the individual patient.

We assume that patients with a targetable mutation (EGFR_classic_, EGFR_non-classic_, ALK, ROS1, BRAF, and NTRK) receive corresponding first-line targeted therapy; all other patients are treated according to PD-L1 expression^1^ (see ESM Table A4).

Second-line treatment in the personalized strategy depends on first-line treatment. For example, patients with an ALK mutation treated with alectinib as first-line treatment are treated with lorlatinib as second-line treatment, and patients with a BRAF mutation treated with a first-line combination of dabrafenib plus trametinib are treated based on PD-L1 expression as second-line treatment. In all other patient subgroups, chemotherapy is given as second-line treatment (ESM Table A5).

To simulate a patient’s disease trajectory under the personalized strategy, the survival models were adjusted to reflect the PFS and OS benefit of receiving targeted therapy or immunotherapy compared to chemotherapy. For patients treated with first-line or second-line targeted therapy, hazard ratios (HR) for PFS derived from RCTs (described in Sect. “Molecular characteristics and effects of novel treatment”) are straightforwardly incorporated in the time-to-event functions from L1T to L2T and from L1T to death and in the time-to-event functions from L2T to L3T and from L2T to death. No treatment adaptations for third-line treatment are currently included in the model. It is known that the long-term benefit of EGFR-TKIs is limited^52^; thus, two approaches were explored for the adaptation of the first-line time-to-event distribution to reflect EGFR-TKI treatment. First, assuming a durable treatment benefit until progression to subsequent treatment-line or death. Second, assuming a limited treatment benefit. A limited treatment benefit was achieved by assuming that from a given time point “t” after start of treatment, the patients’ time to event function to subsequent treatment or death would be identical to the time to event function fitted under the non-personalized treatment strategy. Time point “t” was calibrated against the validation dataset by comparing the modelled progression-free survival estimate assuming durable treatment benefit against the progression-free survival estimate from the validation dataset. Then, “t” was the point at which the modelled progression-free survival curve started to deviate from the curve based on the validation dataset. Furthermore, the prognostic value of the EGFR_classic_^53^ and ALK mutation^54^ were incorporated into the time-to-event functions from L1T to L2T and from L1T to death.

#### Simulating time to event for immunotherapy

Evidence suggests that patients treated with immunotherapy are divided into two subgroups: a subgroup of patients who have long-term benefit and a subgroup of patients with moderate benefit^55–57^. Standard time-to-event distributions may fail to capture these differences and in turn underestimate long-term survival outcomes. To account for that, we assumed that the time-to-event from the start of first-line immunotherapy (i.e., from L1T to L2T and from L1T to death) follows a mixture cure time-to-event distribution^58–60^. This mixture cure distribution was implemented by assuming that 23 percent of patients were long-term survivors (we use the term ‘long-term survivor fraction’ to refer to the cured fraction in the mixture distribution). The long-term survivor fraction was based on the published five-year survival probability from the Keynote-001 trial^56^. We randomly assigned a subgroup of patients to the long-term survivors. These were no longer at risk for lung cancer death after treatment but died from background mortality instead. Patients not pertaining to the group of long-term survivors (moderate survivors) are subjected to the event-specific hazards of the parMSSM with adjusted HR. The input HR (inHR) for immunotherapy in the subgroup of moderate survivor patients required calibration to ensure that the overall HR for all patients receiving immunotherapy compared to chemotherapy (outHR) was equal to what was observed in RCTs (ESM Table A4).

In addition, a deterministic sensitivity analysis (DSA) was performed to assess the impact of assuming a mixture cure time-to-event distribution as well as the impact of the assumed long-term survivor fraction. DSA was performed by assuming different values for the long-term survivor fraction. Assuming a zero percent long-term survivor fraction is equivalent to using a standard time-to-event distribution (here, Gompertz distribution). Value of the long-term survivor fraction were varied between 14 and 34% (corresponding to the 95% confidence interval [95% CI] of the five-year survival rate in Keynote-001^56^) in steps of 5%.

### Validation of the microsimulation model

The microsimulation model was validated according to the ISPOR-SMDM guideline^61^.

#### Internal validity of the microsimulation model

We compared the simulated patient attributes and survival time under the non-personalized treatment strategy with those observed in the template dataset. Model output was based on simulating 1000 runs, each with the same sample size as the template dataset (*2196 patients*). The comparison was performed visually and quantitatively. We visualized the distribution of patients’ attributes and parameters of the regression models. For associations among baseline attributes, we computed the percentage of simulated runs having a bivariate testing p-value smaller than 0.05 and compared it to the observed p-value in the template dataset.

For survival times, we visualized the distribution of simulated survival probabilities at 1 to 60 months for first-line, second-line, third-line PFS and OS and compared them with the survival probabilities from the template dataset. Finally, we evaluated the proportion of simulated medians and means of PFS and OS times that were contained within the respective 95% CIs of the observed medians and means in the template dataset.

#### External validity of the microsimulation model

The adapted model for the simulation of a personalized treatment strategy was externally validated. To perform external validation, we simulated the personalized strategy with 300,000 patients and compared the modelled PFS and OS curves with those of real-world PFS and OS curves. Validated subgroups are described in Sect. “Validation datasets”.

It should be noted that model validation and model building were iterative processes. When validation indicated that the model results were undesirable, the model was adjusted and re-validated again. Where appropriate, we have reported the model results of pre- and post-validation adjustment.

### Ethical statement

All methods were carried out in accordance with relevant guidelines and regulations. The original data collection (Santeon registry 2008–2014) was approved by the Santeon institutional review board, and informed consent was waived (SDB219-008). Data were provided to the authors in a de-identified fashion. The study was performed in accordance with the ethical standards of the institutional and national research committee and with the 1964 Helsinki Declaration and its later amendments or comparable ethical standards.

## Results

### Time to event estimates

For treated patients in the template data, the median time from DIAG to L1T was 1.00 month (95% CI 0.92–1.05), and it was significantly shorter for females than for males. For BSC patients, the median time from DIAG to death was 2.27 months (95% CI 2.14–2.46), and it was significantly shorter for patients with bad or unknown PS compared to good PS and for females compared to males.

The hazard from L1T to L2T was significantly lower for females and decreased with increasing age at diagnosis, while the hazard from L1T to death was significantly higher for bad PS than for good PS. For patients in L2T, their hazard to L3T was significantly lower for females than for males and decreased with increasing age at diagnosis. The hazard from L2T to death was significantly higher for patients treated with carboplatin doublets in first-line than for patients who received cisplatin doublets and was lower for females than for males. The results of the parametric survival analyses for all time-to-event models are given in ESM Table A7.

### Modelled progression-free and overall survival time

The results of the microsimulation model for the non-personalized and personalized treatment strategies are given in Table 1. Out of 300,000 simulated patients, 65 percent received BSC. The mean OS for BSC was 5.0 months irrespective of treatment strategy. For treated patients (35%), the mean OS was 11.2 months and 58.0 months for the non-personalized strategy and the personalized strategy, respectively.

**Table 1:** Modelled median and mean PFS and OS in months (n = 300,000).

Biomarker	1L Treat	n (%)	PFS		OS	
			Median	Mean	Median	Mean
Best-supportive care^a^						
N/A	BSC	195,700 (65.2)	–	–	2.2	5.0
Non-personalized strategy						
ALL Treated	PDCT	104,300 (34.8)	5.4	9.4	6.8	11.2
Personalized strategy						
ALL Treated	–	104,300 (34.8)	11.2	55.4	13.6	57.2
Targeted therapy (TT)						
ALL TT	–	14,860 (14.3)^c^	14.7	25.5	16.8	28.9
EGFR_classic_^b^	EGFR-TKI	7380 (7.1)	14.4	15.9	16.2	20.3
EGFR_non-classic_^b^	Afatinib	1060 (1.0)	14.9	16.4	16.2	18.2
ALK	Alectinib	2100 (2.0)	40.6	88.7	44.0	91.4
ROS1^b^	Crizotinib	1970 (1.9)	13.3	14.9	14.8	16.6
BRAF^b^	Dabraf & Tramet	2190 (2.1)	8.5	12.2	10.9	15.1
NTRK_(1, 2, 3)_^b^	Larotrectinib	160 (0.2)	7.6	10.2	8.8	11.7
Immunotherapy (IT) 23% long-term survivor fraction						
ALL IT	–	89,430 (85.7)	10.5	60.5	12.8	61.9
PD-L1 ≥ 50%	Pembrolizumab	5670 (5.4)	10.9	62.4	13.1	63.8
	Long-term	1310	218	227	218	227
	Moderate	4360	7.0	12.7	8.6	14.4
PD-L1 ≥ 50%	Pembro & PDCT	17,000 (16.3)	17.1	65.1	19.3	66.5
	Long-term	3940	209	219	209	219
	Moderate	13,060	10.9	18.7	12.8	20.6
PD-L1 1–49%	Pembro & PDCT	66,760 (64.0)	9.3	59.1	11.5	60.5
	Long-term	15,350	209	222	209	222
	Moderate	51,410	6.0	10.6	7.5	12.4
Deterministic sensitivity analysis of the mixture cure distribution for IT						
0% long-term survivor fraction						
ALL IT	–	89,430 (85.7)	11.4	19.5	13.4	21.4
PD-L1 ≥ 50%	Pembrolizumab	5670 (5.4)	11.5	19.2	13.6	21.1
PD-L1 ≥ 50%	Pembro & PDCT	17,000 (16.3)	16.6	26.0	18.9	27.9
PD-L1 1–49%	Pembro & PDCT	66,760 (64.0)	10.4	17.9	12.3	19.8

Biomarker, molecular biomarker; 1L treat, first-line systemic treatment; n, number of simulated patients; PFS, progression-free survival; OS, overall survival; BSC, best-supportive care; PDCT, platinum-based doublet chemotherapy; Dabraf & Tramet, combination of dabrafenib with trametinib; Pembro, pembrolizumab; EGFR_classic,_ epidermal growth factor receptor (exon 19 deletions and exon 21 L858R point mutations); EGFR_non-clasic_, epidermal growth factor receptor (non-classic activating EGFR mutations, resistance mutations, and other mutations); ALK, anaplastic lymphoma kinase gene rearrangements; ROS1, genetic aberrations of ROS proto-oncogene 1; BRAF, B-Raf proto-oncogene; NTRK_(1,2,3)_, neurotrophic receptor tyrosine kinase 1; PD-L1, high programmed death ligand 1 expression; Long-term, long-term survivors fraction; Moderate, moderate survivors fraction.

^a^Best-supportive care group was kept constant in both strategies.

^b^It is assumed that first-line treatment benefits wear-out after 15 months.

^c^Percentage within subgroups of personalized strategy were calculated out of the total number of patients treated (denominator 104,300).

For treated patients in the personalized strategy, 14 percent received first-line targeted therapy, while 86 percent received immunotherapy. The mean OS was 28.9 months and 61.9 months for targeted therapy and for immunotherapy, respectively. The molecular subgroups with the highest mean OS were patients with an ALK mutations who were treated with alectinib (mean OS of 91.4 months, i.e. 7.6 years). This was followed by patients with PD-L1 ≥ 50% who were treated with a combination of pembrolizumab plus chemotherapy (mean OS of 66.5 months, i.e. 5.5 years).

### Internal validation of microsimulation model

Patients’ baseline attributes as well as the parameters of the baseline regression models from 1000 runs of the microsimulation model were distributed well around corresponding values from the template dataset (ESM Figs. A1 and A2). Likewise, the modelled bivariate associations were in agreement with the results from the template dataset (ESM Table A2 and Fig A1).

**Figure 3:**
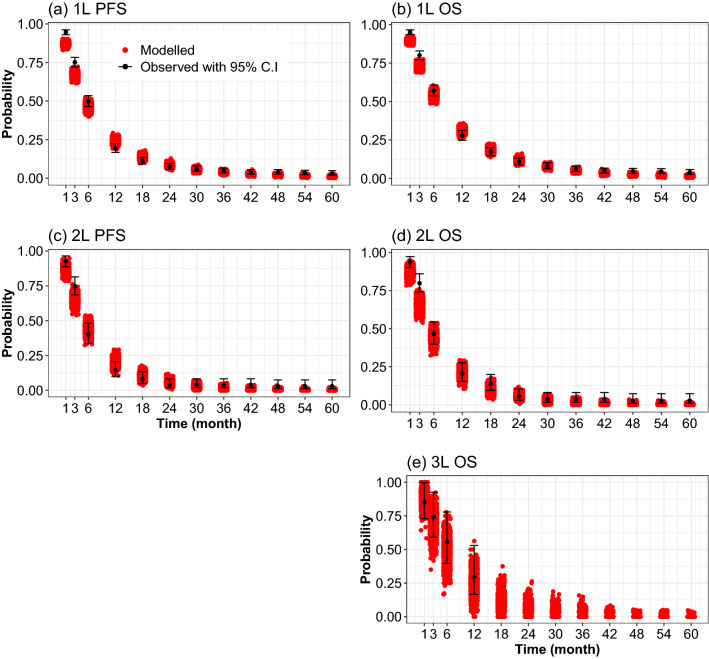
Jitter plot comparing modelled and observed progression-free survival (PFS) and overall survival (OS) probabilities. (**a** and **b**), PFS and OS from the start of first-line treatment (1L); (**c**) and (**d**), PFS and OS from the start of second-line treatment (2L); (**e**), OS from the start of third-line treatment (3L). The red dots (modelled) refer to the simulated values for a non-personalized treatment strategy where simulated patients are treated with a first-line platinum-based chemotherapy as in the template dataset. The black dots with 95% C.I indicate the point estimates and 95% confidence intervals of observed probabilities from the template dataset (Santeon registry 2008–2014).

The distribution of the PFS and OS probabilities at specific time points of 1000 simulation runs for the non-personalized strategy are given in Fig. 3a–e. Except for the tails of the distributions, simulated PFS and OS probabilities from the first-line, second-line and third line were distributed around the observed values largely overlapping the respective 95% CI. Likewise, the simulated medians, restricted means and proportion of patients who progressed to L2T and L3T matched acceptably well with the observed 95% CI (ESM Fig. A3).

### External validation

**Figure 4:**
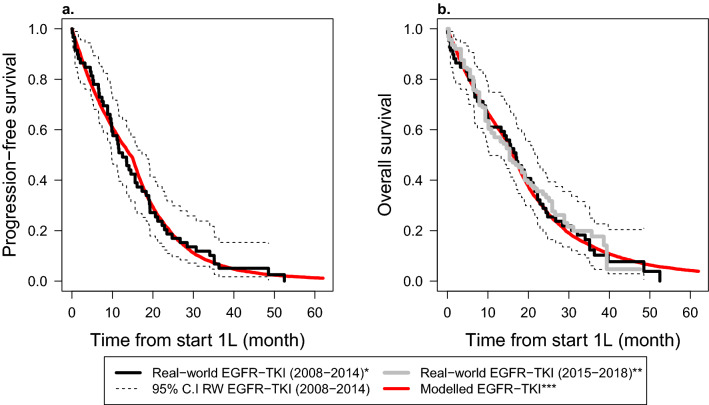
Comparison of modelled and real-world progression-free survival (**a**) and overall survival (**b**) curves for patients with epidermal growth factor receptor (EGFR) mutations and treated with a first-line EGFR tyrosine kinase inhibitors (EGFR-TKIs). EGFR-TKI were gefitinib or erlotinib; C.I, confidence interval; RW, Real-world; 1L, first-line systemic treatment. *The real-world data of 52 patients diagnosed and treated between 2008 and 2014 in Santeon hospitals^42^. **The reconstructed (digitized) real-world overall survival (OS) data of 147 patients treated between 2015 and 2018 in Santeon hospitals. The OS data were digitized from the curve published by Cramer-van der Welle et al., 2021^19^. Progression-free survival (PFS) data was not available. ***Modelled data was simulated assuming the hazard ratios of 0.43 and 0.36 for gefitinib and erlotinib compared to chemotherapy, respectively^39^, and hazard ratio of 0.82 for prognostic value of EGFR positive compared to EGFR negative^40^. EGFR-TKI benefit was assumed to wear out after 15 months.

The validation results for the EGFR_classic_ subgroup who were treated with first-line TKI (gefitinib or erlotinib) are given in Fig. 4. When the first-line TKI benefit was restricted to 15 months, the modelled PFS and OS curves matched well to the real-world curves of the Santeon registry 2008–2014 cohort. Similarly, the modelled OS curve matched well with the real-world curve of the Santeon registry 2015–2018 cohort. Modelled median PFS of 14.4 months was contained within the 95% CI of the median PFS from the Santeon registry 2008–2014 (i.e., 12.5, 95% CI 9.8–18.3 months). Similarly, the modelled median OS of 16.2 months was contained within the 95% CI of the median OS from the Santeon registry 2008–2014 and the Santeon registry 2015–2018, that is, 17 months (95% CI 10.3–22.2) and 15.5 months (95% CI 11.6–19.1), respectively. It should be noted that when the first-line TKI benefit was assumed to last until progression, the modelled median PFS and OS were not affected, but the long-term PFS and OS were overestimated compared to what was observed (ESM Fig. A4).

**Figure 5:**
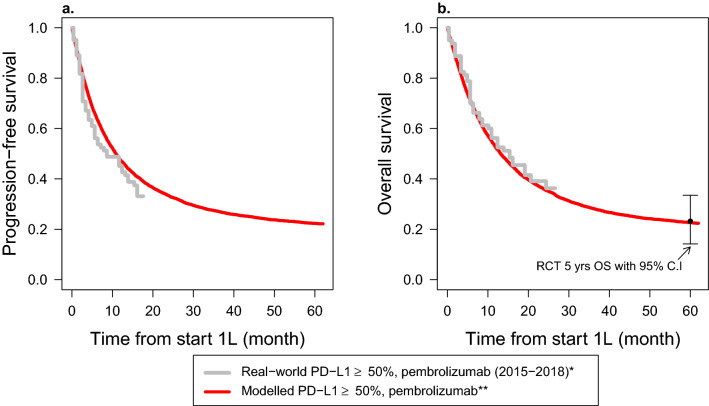
Comparison of modelled and real-world progression-free survival (**a**) and overall survival (**b**) curves for patients with high programmed death ligand 1 (PD-L1 ≥ 50%) expression and treated with first-line pembrolizumab monotherapy. *Reconstructed (digitized) progression-free and overall survival data of 83 patients diagnosed and treated between 2015 and 2018 in Santeon hospitals^19^. **The modelled data was simulated assuming a hazard ratio of 0.5 for pembrolizumab compared to chemotherapy^38^ and a long-term survivor fraction of 23 percent^56^. The black dot and interval bar (RCT 5-yr OS with 95% C.I) indicates a five-year overall survival with 95% confidence interval from Keynote-001^56^.

Figure 5 and ESM Table A8 show the validation results for the PD-L1 ≥ 50% subgroup who were treated with first-line pembrolizumab monotherapy. Figure 5 shows that the modelled PFS and OS curves match well with the real-world curve of Santeon registry 2015–2018 (the median lies within the 95% C.I of Santeon registry 2015–2018). Beyond 12 months, the model seems a bit optimistic with respect to PFS but matches well with the OS. Furthermore, the modelled 24-month and 36-month OS rates were close to the US real-world survival rates^21^ (ESM Table A8). The 60-month (5-year) survival rate was the same as that of Keynote-001^56^, which was 23%.

The DSA results for the assumption of a mixture cure time-to-event distribution and the value of the long-term survivor fraction are given in Table 1 and ESM Fig. A5. ESM Fig. A5 shows that the long-term survivor fraction mainly impacts the long-term PFS and OS. The OS proportion at five years ranged from 16 to 31% when the long-term survivor fraction was varied between 14 and 34%. When a standard time-to-event distribution was assumed (i.e., 0 percent long-term survivor fraction) in patients with PD-L1 ≥ 50% who were treated with pembrolizumab, the median OS was increased by 0.5 months, while the mean OS decreased by 42.7 months (3.6 years, Table 1). The large impact on mean OS was due to the long survival time in the long-term survivor subgroup, which had a mean OS of 222 months (18.5 years), while the moderate survivor subgroup had a mean OS of 14.4 months (1.2 years) (Table 1).

Similar findings were obtained for validation of the group of patients who were treated with a combination of pembrolizumab plus chemotherapy and had either PD-L1 ≥ 50% or PD-L1 1–49%. For validation of these two subgroups, we used published US real-world estimates^17,21^ (ESM Table A8).

## Discussion

We developed a patient-level microsimulation model to simulate treatment trajectories of patients with advanced (inoperable) non-squamous NSCLC in the Netherlands. All patients are simulated from diagnosis to death and undergo at most three treatment lines. The model can be used to carry out a range of HTA evaluations in the treatment of non-squamous NSCLC, such as health-economic evaluation, budget impact assessment, or the evaluation of clinical guidelines. It has been argued that multiuse models will improve efficiency and consistency in decision making^62^. In the near future, we plan to use the model to perform an early cost-effectiveness analysis (CEA) of using whole-genome sequencing in the treatment decision of NSCLC patients treated with immunotherapy in the Netherlands.

Internal validation as well as external validation for specific subgroups was demonstrated. External validation was demonstrated for the subgroups of patients with an EGFR_classic_ mutation treated with first-line TKI and patients with PD-L1 ≥ 50% treated with immunotherapy. External validation was performed comparing the results of a personalized treatment strategy against the real-world PFS and OS data.

For the EGFR_classic_ subgroup treated with first-line EGFR-TKIs, the model overestimated long-term PFS and OS compared to the real-world data when we assumed EGFR-TKI benefit to last until progression. When we restricted the first-line EGFR-TKI benefit to up to 15 months, the modelled PFS and OS matched well with the real-world data. Because treatment resistance is known for earlier generations of targeted therapies^52^, we have extended the assumption of limited first-line targeted therapy benefit (up to 15 months) to all patients who received first-line targeted therapies. An exception was made for patients in the ALK subgroup who received a second-generation ALK inhibitor (alectinib) because the durability of the benefit of first-line alectinib has been demonstrated in updated results of the ALEX study^63^.

Immunotherapy is known to have a delayed treatment effect. Also, there is a subgroup of patients who have a better treatment response^58,64–66^. The mixture cure time-to-event distribution (mixture cure model) has been proposed and used as a modelling solution to improve long-term projections for immunotherapy^58–60,67,68^. We have shown that under the standard time-to-event distribution, the model resulted in a lower long-term OS estimate compared to the real-world OS estimates. In contrast, when a mixture cure time-to-event distribution was used, modelled long-term OS estimates were comparable to real-world estimates. Assuming a mixture cure model had almost no impact on median PFS and OS but dramatically increased mean PFS and OS. This increase was caused by the fact that the subgroup of long-term survivors was assumed to have the same mortality rate as the general Dutch population. This latter assumption remains to be validated. Nevertheless, recent updated RCT literature has shown that the subgroup of patients who completed 35 cycles of pembrolizumab (20 to 30 percent of patients) had most treatment benefit with more than 80 percent five-year overall survival rates^55,57^.

HTA decision models for advanced (inoperable) non-squamous NSCLC have been developed previously^10,13,15^. These studies were mainly based on RCTs. We compared the findings of the base-case analysis of these three studies against the results of the personalized strategy of our proposed model. These studies were selected because they are similar to the proposed model in terms of modelling a Dutch perspective and/or used similar sources to incorporate the benefits of novel treatments.

The mean life years (mean OS) of the currently proposed model for the EGFR-TKI subgroup is four months lower than the mean life years reported by Holleman et al.^10^, that is, 20.3 months in the proposed model compared to 24 (25) months for gefitinib (erlotinib) treatment in Holleman et al. The difference can partly be explained by limiting the benefit of gefitinib (erlotinib) to 15 months to correct for treatment resistance, as also demonstrated in the real-world data. Meanwhile, for the ALK subgroup treated with alectinib, a durable treatment benefit was assumed, resulting in a mean number of life years twice as high in our proposed model compared to the mean life years reported by Simons et al.^13^ (Strategy A, online supplementary), that is, 91.4 months versus 45.6 months. The durability of the treatment benefit of alectinib in patients with an ALK mutation was supported by updated results of the ALEX study^63^.

For patients with PD-L1 ≥ 50% who were treated with pembrolizumab monotherapy, the proposed model with a 23% long-term survivor fraction^56^ had twice the mean life years (63.8 months) compared to the reported results by Chouaid et al.^15^ (34 months) as well as by Simons et al.^13^ (29 months). However, when a zero percent long-term survivor fraction was assumed, our proposed model had lower mean life years (21.1 months) compared to Chouaid et al. and Simons et al. Similarly, for patients with PD-L1 ≥ 50% treated with a combination of pembrolizumab and chemotherapy, the mean life years were three times higher compared to Simons et al. when the long-term survivor fraction was assumed (66.5 months vs. 23 months) and approximately 4 months higher when the long-term survivor fraction was not assumed (27.9 months vs. 23 months).

The differences between the currently proposed model and the three models mentioned above are partly due to different modeling strategies, that is, a microsimulation DES model versus Markov cohort models^10,13^ and a partitioned survival model^15^, as well as different modelling assumptions adopted in the proposed model, such as assuming the mixture cure time-to-event distribution for immunotherapy. However, this difference probably also partly due to the different underlying populations. For our proposed model, the real-world population was used instead of RCTs. To reproduce real-world PFS and OS curves under a personalized strategy, some model assumptions had to be made that were not implemented in the other models.

Our model has a number of strengths. First, the proposed model accounted for baseline patient heterogeneity by including patient characteristics such as performance status, gender, and age.

Second, in our model, we included patients who received best supportive care (BSC) after diagnosis. It is uncommon for HTA studies to include the BSC subgroup; nevertheless, BSCs remain the largest subgroup of advanced (inoperable) non-squamous NSCLC. In the Netherlands, approximately 50% of patients with advanced NSCLC do not start a first-line treatment^19^. Ignoring this subgroup when making projections of the long-term benefits, costs, and budget impact of personalized care for NSCLC in a specific setting may lead to a distorted result. In addition, the proportion of patients undergoing systemic treatment with life-prolonging intent may change over time as a result of improved toxicity profiles for certain personalized treatments, such as immunotherapies. The potential impact of including or ignoring BSC in the Netherlands can indirectly be identified from Cramer-van der Welle et al.^18^ and Cramer-van der Welle et al.^19^. In Cramer-van der Welle et al.^18^, the proportion of patients receiving BSC after diagnosis was 59% (out of 2989 patients diagnosed between 2008 and 2014), while in Cramer-van der Welle et al.^19^, the proportion of patients with BSC dropped to 48% (out of 1950 patients diagnosed between 2015 and 2018).

Third, the proposed model has the flexibility to allow different modelling assumptions to be evaluated using the same model by simply changing the model’s arguments. For example, the proposed model allows us to model both limited and durable treatment benefit assumptions for targeted therapies. Likewise, for immunotherapy, both the standard time-to-event distribution and the mixture cure time-to-event distribution can be assumed by specifying model arguments. Thus, the impact of such assumptions on life years and cost can be evaluated using one model.

Furthermore, the model is syntax based and programmed in the R computing language^48^. A syntax-based model increases transparency, reproducibility^46^ and flexibility. Thus, it can easily be adapted or extended to future data or future developments, if needed.

Our model has a number of limitations. First, we assumed no post-progression survival benefit of treatment. This means that a patient’s survival after first-line progression is independent of treatment regimens received during the first-line treatment. This assumption may under- or overestimate the estimated long-term survival benefit of novel treatments such as EGFR-TKIs and pembrolizumab. Nevertheless, there is limited evidence for the potential magnitude and direction of such benefit. In addition, we only accounted for a prognostic impact of the presence of an EGFR_classic_^53^ mutation and the presence of ALK gene rearrangements^54^. For the remaining molecular subgroups, we did not have evidence of their prognostic values. However, as the model is flexibly programmed, such prognostic impact can be included when evidence becomes available, and this also holds for a post-progression treatment benefit.

Second, there are currently no phase III RCTs of crizotinib for patients with a ROS1 mutation, the combination of dabrafenib and trametinib for patients with a BRAF_v600E_ mutation, and larotrectinib for patients with an NTRK_(1, 2, 3)_ mutation. In simulating a personalized treatment strategy for the subgroups ROS1, BRAF_v600E_, and NTRK_(1, 2, 3)_, it was assumed that crizotinib in ROS1 has similar effectiveness as crizotinib in the ALK subgroup^35^, dabrafenib and trametinib were equally effective in NSCLC BRAF_v600E_ as in melanoma BRAF_val600_^69^, and larotrectinib in NTRK_(1, 2, 3), it_ was assumed to have a PFS HR similar to that of the prognostic value of EGFR_classic_^40^. There were no data to validate the model outputs of these subgroups. In the future application of the model, a sensitivity analysis surrounding the treatment effectiveness in the mentioned subgroups will be necessary.

Third, we assumed that 23% of patients treated with first-line immunotherapy (pembrolizumab monotherapy or in combination with chemotherapy) were long-term survivors with survival matching the survival of the general population given age and sex (background mortality). Patients in the long-term survival group were not eligible for second-line treatment, and they died from other causes after first-line treatment. The long-term survivor fraction of 23% was based on patients attaining five-year survival in keynote-001^56^, which is supported by the long-term follow-up of keynote-024^57^. Pembrolizumab treatment is relatively new in clinical care; therefore, we have no real-world data to validate the fraction of long-term survivors nor their survival beyond 36 months. Through sensitivity analysis, the impact of the long-term survivor fraction was explored.

Fourth, the real-world template dataset (Santeon registry 2008–2014) that formed the basis of our model had a cohort of patients diagnosed and treated prior to the era of personalized treatment. Thus, the template data set unfortunately did not contain molecular characteristics or the currently used novel treatments. To simulate a personalized strategy, we incorporated molecular information from the literature, as well as the impact of novel treatment compared to standard chemotherapy based on published RCTs. This constrained us to the use of parametric PH distributions (Exponential, Weibull, and Gompertz) to describe the transitions from the start of first-line treatment to death and necessitated the assumption that novel treatments have the same effect on competing transitions (i.e., time to subsequent treatment line or to death).

Fifth, we had limited data for external validation of the model. For the personalized strategy, only the subgroup of patients with an EGFR mutation treated with TKIs and the subgroup of patients with PD-L1 ≥ 50% treated with pembrolizumab monotherapy were externally validated with Dutch real-world data^19,42^. For the non-personalized strategy, the model was not validated externally. The subgroup of patients treated with first-line chemotherapy in Santeon hospitals between 2015 and 2018^19^ is not directly comparable to the simulated patients in the non-personalized strategy. The 2015–2018 dataset includes patients with both squamous and no-squamous histology and about a quarter of patients received subsequent immunotherapy (mainly nivolumab), while the simulated non-personalized strategy in our presented model simulates patients with non-squamous histology treated with chemotherapy in all treatment lines.

Last, in the presence of competing events, we have used cause-specific hazard functions to sample the time of each competing event separately and subsequently select the event that occurred first (in the HTA literature, this method is termed event-specific distribution (ESD)^24^). This choice was a deviation from the standard recommendation to jointly estimate the event time and select the event in a second step by using (multinomial) logistic regression^24,70^. In this situation, we preferred ESD because of its convenience in estimation in the presence of censoring. Additionally, if a novel treatment is believed to have a different impact on the hazard rate of two competing events, ESD can accommodate this by appropriately adjusting the HRs of particular transitions.

## Conclusion

We developed a multi-application microsimulation model for advanced (inoperable) non-squamous NSCLC in the Netherlands using real-world data. The model was populated with real-world data from six large teaching hospitals in the Netherlands. The model was internally validated and externally validated for the EGFR subgroup of NSCLC patients and for patients with PD-L1 expression receiving immunotherapy. We can argue that, being based on real-world data, the presented model is suitable to project long-term outcomes and cost-effectiveness of novel diagnostic-treatment combinations in the Dutch setting and is consequently suited to inform Dutch policy makers. In the near future, the model will be used to perform an early CEA of using whole-genome sequencing in immunotherapy decision in NSCLC in the Netherlands.

## Supplementary Information

Supplementary Information.

## Data Availability

Correspondence and requests for data and materials should be addressed to Veerle M.H. Coupé.

## References

[1] Planchard D (2018). Metastatic non-small cell lung cancer: ESMO clinical practice guidelines for diagnosis, treatment and follow-up. Ann. Oncol..

[2] National Comprehensive Cancer Network. Non-Small Cell Lung Cancer (version 3.2020). (2020).

[3] van den Broek D (2019). Implementation of novel molecular biomarkers for non-small cell lung cancer in the netherlands: How to deal with increasing complexity. Front. Oncol..

[4] Arbour KC, Riely GJ (2019). Systemic therapy for locally advanced and metastatic non-small cell lung cancer: A review. JAMA.

[5] Nesline MK, Knight T, Colman S, Patel K (2020). Economic burden of checkpoint inhibitor immunotherapy for the treatment of non-small cell lung cancer in us clinical practice. Clin. Ther..

[6] Caro JJ, Briggs AH, Siebert U, Kuntz KM, Force I-SMGRPT (2012). Modeling good research practices–overview: A report of the ISPOR-SMDM modeling good research practices task force–1. Value Health.

[7] National Institute for Health and Care Excellence. Guide to the methods of technology apprisal [NICE Guideline No. 9]. (2013). 27905712

[8] Krijkamp EM (2018). Microsimulation modeling for health decision sciences using R: A tutorial. Med. Decis. Making.

[9] Weinstein MC (2003). Principles of good practice for decision analytic modeling in health-care evaluation: Report of the ISPOR task force on good research practices-modeling studies. Value Health.

[10] Holleman MS, Al MJ, Zaim R, Groen HJM, Uyl-de Groot CA (2020). Cost-effectiveness analysis of the first-line EGFR-TKIs in patients with non-small cell lung cancer harbouring EGFR mutations. Eur. J. Health Econ..

[11] Barbier MC (2021). A cost-effectiveness analysis of pembrolizumab with or without chemotherapy for the treatment of patients with metastatic, non-squamous non-small cell lung cancer and high PD-L1 expression in Switzerland. Eur. J. Health Econ..

[12] Westwood M (2014). Epidermal growth factor receptor tyrosine kinase (EGFR-TK) mutation testing in adults with locally advanced or metastatic non-small cell lung cancer: A systematic review and cost-effectiveness analysis. Health Technol. Assess.

[13] Simons M (2021). Early cost effectiveness of whole-genome sequencing as a clinical diagnostic test for patients with inoperable stage IIIB C/IV non-squamous non-small-cell lung cancer. Pharmacoeconomics.

[14] van Amerongen RA (2016). Next-generation sequencing in NSCLC and melanoma patients: A cost and budget impact analysis. Ecancermedicalscience.

[15] Chouaid C (2019). Cost-effectiveness analysis of pembrolizumab versus standard-of-care chemotherapy for first-line treatment of PD-L1 positive (>50%) metastatic squamous and non-squamous non-small cell lung cancer in France. Lung Cancer.

[16] Pasello G (2020). Real world data in the era of immune checkpoint inhibitors (ICIs): Increasing evidence and future applications in lung cancer. Cancer Treat. Rev..

[17] Velcheti, V., Hu, X. H., Piperdi, B. & Burke, T. Real-world outcomes of first-line pembrolizumab plus pemetrexed-carboplatin for metastatic nonsquamous NSCLC at US oncology practices. *Sci. Rep.-UK* **11**, doi:ARTN 922210.1038/s41598-021-88453-8 (2021). 10.1038/s41598-021-88453-8 PMC8080779 33911121

[18] Cramer-van der Welle CM (2018). Systematic evaluation of the efficacy-effectiveness gap of systemic treatments in metastatic nonsmall cell lung cancer. Eur. Respir. J..

[19] Cramer-van der Welle CM (2021). Real-world outcomes versus clinical trial results of immunotherapy in stage IV non-small cell lung cancer (NSCLC) in the Netherlands. Sci. Rep..

[20] Kehl KL, Greenwald S, Chamoun NG, Manberg PJ, Schrag D (2021). Association between first-line immune checkpoint inhibition and survival for medicare-insured patients with advanced non-small cell lung cancer. JAMA Netw. Open.

[21] Waterhouse D (2021). Real-world outcomes of immunotherapy-based regimens in first-line advanced non-small cell lung cancer. Lung Cancer.

[22] Pouwels X (2020). An economic evaluation of eribulin for advanced breast cancer treatment based on the Southeast Netherlands advanced breast cancer registry. Acta. Oncol..

[23] Davis, S., Stevenson, M., Tappenden, P. & Wailoo, A. NICE DSU technical support document 15: Cost-effectiveness modelling using patient-level simulation, (2014). 27466644

[24] Degeling K, Koffijberg H, Franken MD, Koopman M, MJ IJ (2019). Comparing strategies for modeling competing risks in discrete-event simulations: A simulation study and illustration in colorectal cancer. Med. Decis. Making.

[25] Okunade, O., Arora, J., Haverhals, A. & Niessen, L. Collaborating for value: The Santeon Hospitals in the Netherlands. (2017). <https://ichom.org>.

[26] D'Addario, G., Felip, E. & Group, E. G. W (2008). Non-small-cell lung cancer: ESMO clinical recommendations for diagnosis, treatment and follow-up. Ann. Oncol..

[27] D'Addario G (2010). Metastatic non-small-cell lung cancer: ESMO clinical practice guidelines for diagnosis, treatment and follow-up. Ann. Oncol..

[28] Peters S (2012). Metastatic non-small-cell lung cancer (NSCLC): ESMO clinical practice guidelines for diagnosis, treatment and follow-up. Ann. Oncol..

[29] Reck M (2014). Metastatic non-small-cell lung cancer (NSCLC): ESMO clinical practice guidelines for diagnosis, treatment and follow-up. Ann Oncol.

[30] Jordan EJ (2017). Prospective comprehensive molecular characterization of lung adenocarcinomas for efficient patient matching to approved and emerging therapies. Cancer Discov..

[31] Kuijpers C (2018). Association of molecular status and metastatic organs at diagnosis in patients with stage IV non-squamous non-small cell lung cancer. Lung Cancer.

[32] Dietel M (2019). Real-world prevalence of programmed death ligand 1 expression in locally advanced or metastatic non-small-cell lung cancer: The global, multicenter EXPRESS study. Lung Cancer.

[33] Forsythe A (2020). A systematic review and meta-analysis of neurotrophic tyrosine receptor kinase gene fusion frequencies in solid tumors. Ther. Adv. Med. Oncol..

[34] Peters S (2017). Alectinib versus crizotinib in untreated alk-positive non-small-cell lung cancer. N. Engl. J. Med..

[35] Solomon BJ (2014). First-line crizotinib versus chemotherapy in ALK-positive lung cancer. N. Engl. J. Med..

[36] Gandhi L (2018). Pembrolizumab plus chemotherapy in metastatic non-small-cell lung cancer. N. Engl. J. Med..

[37] Herbst RS (2016). Pembrolizumab versus docetaxel for previously treated, PD-L1-positive, advanced non-small-cell lung cancer (KEYNOTE-010): A randomised controlled trial. Lancet.

[38] Reck M (2016). Pembrolizumab versus chemotherapy for PD-L1-positive non-small-cell lung cancer. N. Engl. J. Med..

[39] Holleman MS, van Tinteren H, Groen HJ, Al MJ, Uyl-de Groot CA (2019). First-line tyrosine kinase inhibitors in EGFR mutation-positive non-small-cell lung cancer: A network meta-analysis. Onco Targets Ther..

[40] Simons M (2020). Observed versus modelled lifetime overall survival of targeted therapies and immunotherapies for advanced non-small cell lung cancer patients–a systematic review. Crit. Rev. Oncol. Hematol..

[41] Hoyle MW, Henley W (2011). Improved curve fits to summary survival data: Application to economic evaluation of health technologies. BMC Med. Res. Methodol..

[42] Sluga R (2018). Utilization of molecular testing and survival outcomes of treatment with first- or second-line tyrosine kinase inhibitors in advanced non-small cell lung cancer in a Dutch population. Anticancer Res.

[43] Latimer, N. NICE DSU technical support document 14: Undertaking survival analysis for economic evaluations alongside clinical trials–extrapolation with patient-level data. (2011). 27905716

[44] Ishak KJ, Kreif N, Benedict A, Muszbek N (2013). Overview of parametric survival analysis for health-economic applications. Pharmacoeconomics.

[45] Putter H, Fiocco M, Geskus RB (2007). Tutorial in biostatistics: Competing risks and multi-state models. Stat. Med..

[46] Williams C, Lewsey JD, Briggs AH, Mackay DF (2017). Cost-effectiveness analysis in R using a multi-state modeling survival analysis framework: A tutorial. Med. Decis. Making.

[47] Wolff HB (2020). Cost-effectiveness of stereotactic body radiation therapy versus video assisted thoracic surgery in medically operable stage I non-small cell lung cancer: A modeling study. Lung Cancer.

[48] R: A language and environment for statistical computing (R Foundation for Statistical Computing, Vienna, Austria, 2020).

[49] de Wreede LC, Fiocco M, Putter H (2010). The mstate package for estimation and prediction in non- and semi-parametric multi-state and competing risks models. Comput. Methods Programs Biomed..

[50] eha: Event History Analysis. R package version 2.8.1 (CRAN, 2020).

[51] Jackson CH (2016). flexsurv: A platform for parametric survival modeling in R. J. Stat. Softw..

[52] Kohler J, Schuler M (2013). Afatinib, erlotinib and gefitinib in the first-line therapy of EGFR mutation-positive lung adenocarcinoma: A review. Onkologie.

[53] Fang S, Wang Z (2014). EGFR mutations as a prognostic and predictive marker in non-small-cell lung cancer. Drug. Des. Devel. Ther..

[54] Wang ZL (2017). Anaplastic lymphoma kinase gene rearrangement predicts better prognosis in NSCLC patients: A meta-analysis. Lung Cancer.

[55] Awad MM (2021). Long-term overall survival from KEYNOTE-021 cohort G: Pemetrexed and carboplatin with or without pembrolizumab as first-line therapy for advanced nonsquamous NSCLC. J. Thorac. Oncol..

[56] Garon EB (2019). Five-year overall survival for patients with advanced nonsmall-cell lung cancer treated with pembrolizumab: Results from the phase I KEYNOTE-001 study. J. Clin. Oncol..

[57] Reck M (2021). Five-year outcomes with pembrolizumab versus chemotherapy for metastatic non-small-cell lung cancer with PD-L1 tumor proportion score >/= 50. J. Clin. Oncol..

[58] Bullement A, Latimer NR, Bell Gorrod H (2019). Survival extrapolation in cancer immunotherapy: A validation-based case study. Value Health.

[59] Othus M, Bansal A, Koepl L, Wagner S, Ramsey S (2017). Accounting for cured patients in cost-effectiveness analysis. Value Health.

[60] To YH (2021). Circulating tumour DNA as a potential cost-effective biomarker to reduce adjuvant chemotherapy overtreatment in stage II colorectal cancer. Pharmacoeconomics.

[61] Eddy DM (2012). Model transparency and validation: A report of the ISPOR-SMDM modeling good research practices task force–7. Value Health.

[62] Feenstra, T. *et al.* Multi-use disease models: A blueprint for application in support of health care insurance coverage policy and a case study in Diabetes Mellitus. (2020). <https://rivm.openrepository.com/bitstream/handle/10029/623093/2018-0145.pdf?sequence=1>.

[63] Mok T (2020). Updated overall survival and final progression-free survival data for patients with treatment-naive advanced ALK-positive non-small-cell lung cancer in the ALEX study. Ann. Oncol..

[64] Ferrara R (2018). Do immune checkpoint inhibitors need new studies methodology?. J. Thorac. Dis..

[65] Buyse M, Saad ED, Burzykowski T, Peron J (2020). Assessing treatment benefit in immuno-oncology. Stat. Biosci..

[66] Huang B, Ting N (2020). Introduction to special issue on statistical methods for cancer immunotherapy. Stat. Biosci..

[67] Gibson E (2017). Modelling the survival outcomes of immuno-oncology drugs in economic evaluations: A systematic approach to data analysis and extrapolation. Pharmacoeconomics.

[68] Ouwens M (2019). Estimating lifetime benefits associated with immuno-oncology therapies: Challenges and approaches for overall survival extrapolations. Pharmacoeconomics.

[69] Long GV (2015). Dabrafenib and trametinib versus dabrafenib and placebo for Val600 BRAF-mutant melanoma: A multicentre, double-blind, phase 3 randomised controlled trial. Lancet.

[70] Karnon J (2012). Modeling using discrete event simulation: a report of the ISPOR-SMDM modeling good research practices task force–4. Value Health.

