# Supplementary material for: Development and validation of a decision model for the evaluation of novel lung cancer treatments in the Netherlands
Source: Sci Rep. 2023 Feb 9;13:2349. doi: 10.1038/s41598-023-29286-5 (PMC9911639; doi:10.1038/s41598-023-29286-5)
Supplement: Supplementary file 1 — Supplementary Information. [file 41598_2023_29286_MOESM1_ESM.pdf]

ELECTRONIC SUPPLIMENTARY MATRIALS

Development and validation of a decision model for the evaluation of novel lung cancer treatments in the Netherlands.

AUTHORS

Zakile A. Mfumbilwa,[1] Janneke A. Wilschut,[1] Martijn J.H.G. Simons,[2,3] Bram Ramaekers,[2,3] Manuela Joore,[2,3] Valesca Retèl,[4] Christine M. Cramer-van der Welle,[5] Franz M.N.H. Schramel,[5] Ewoudt M.W. van de Garde,[6,7] and Veerle M.H. Coupé [1*]

AUTHOR AFFILIATION

[1] Department of Epidemiology and Data Science, Amsterdam Public Health, Amsterdam UMC, Vrije Universiteit Amsterdam, de Boelelaan 1117, Amsterdam, the Netherlands.
[2] Department of Clinical Epidemiology and Medical Technology Assessment, Maastricht University Medical Centre+, Maastricht, the Netherlands.
[3] Maastricht University, Care And Public Health Research Institute (CAPHRI), Maastricht, the Netherlands.
[4] Department of Health technology and Services research, University of Twente, Enschede, the Netherlands.
[5] Santeon Hospital Group, Santeon, Herculesplein 38, 3584 AA Utrecht, the Netherlands.
[6] Division of Pharmacoepidemiology and Clinical Pharmacology, Department of Pharmaceutical Sciences, Utrecht University, Utrecht, the Netherlands
[7] Department of Clinical Pharmacy, St. Antonius Hospital, Utrecht/Nieuwegein, the Netherlands

[*] **Address correspondence to** Veerle M.H. Coupé, Department of Epidemiology and Data Science, Amsterdam UMC, PO Box 7057, 1007 MB, Amsterdam, the Netherlands.
Email address: v.coupe@amsterdamumc.nl (Coupé, V.M.H.)

A.1 Methods

Fitting of a parametric multistate statistical model (parMSSM)

The fitting of parametric multistate-statistical model (parMSSM) is carried out as a series of nested competing risk models accounting for competing events (e.g., from L1T to L2T vs from L1T to Death) and delayed entry (left truncation) for in-transit events (i.e., from L2T to L3 and from L3T to Death) [1,2].

For each possible time to event (from event i to event j), a cause-specific hazard model is defined as follows:

$$h_{ij}(t) = h_{0j}(t)\exp(\beta_k^T X_{kl}) \qquad \text{equation (A1)}$$

where $h_{0j}(t)$ is a time dependent baseline hazard of moving from event i to event j. $h_{ij}(t)$ is the hazard function of moving from event i to event j at time t. β_k^T is the transposed vector of model coefficients associated with covariate X_{kl}, with l = 1, 2, … n_{ij}. n_{ij} – total number of patients at risk at t = 0 for moving from event i to event j.

Equation (A1) can be fitted using a parametric or non-parametric framework, and fitting goes as follow;

a) For fitting a specific event-time distribution, patients who experience a competing event are censored at the time of the competing event.
b) For event time from in-transit events (from L2T to L3T, from L2T to Death, and from L3T to Death), patients are not at risk for these movement until they have progressed to event L2T or L3T, respectively.

For a parametric framework, a specification of the distribution function of the baseline hazard $h_{0ij}(t)$ for all time to events is required [3]. In this study we restricted ourselves to three proportional hazard (PH) distribution functions i.e., Exponential, Weibull, and Gompertz. Based on visual inspection, gompertz distribution with monotonic hazard was the best distribution for time from L1T to L2T and L1T to Death, exponential distribution gives the best fit for time from L2T to L3T and L2T to Death. Because of the limited number of patients at risk for time from L3T to Death, an exponential distribution was chosen. Covariates (baseline attributes) for each time-to-event model were selected based on backward selection (cutoff p-value < 0.05).

Construction of the microsimulation model: non-personalized strategy

The microsimulation model was implemented as follow; Patients' profiles were sampled according to the distributions, associations, and parameters described in the methods Sect. "Parameterization of the non-personalized strategy". First, we sampled baseline characteristics (attributes); gender, year of diagnosis, age at diagnosis, CCI, PS. Later, conditional on baseline attributes, we sampled the treatment decision (BSC or treated). For treated patients, the type of systemic chemotherapy (cisplatin containing doublets or carboplatin containing doublets) was sampled taking into account the baseline attributes. Distributions and parameters used to sample patients' baseline attributes and treatment decisions are given in Table A6.

Under the non-personalized strategy; for patients who progressed to second- and third-line chemotherapy. In the second and third lines we assumed that patients have equal prognosis regardless of the type of chemotherapy. Therefore, in the second and third lines, there is no specification of chemotherapy regimens.

Lastly, patients' disease pathways were sampled conditional upon patients' attributes and treatment decisions. Time to event was drawn from the fitted parametric distributions and background mortality as described in Sect. "Parameterization of the non-personalized strategy". For competing events, the event with the shortest event time was taken as the event that occurred for that patient. At any disease event, patients could die due to other causes (DoC) described by background mortality. The process was carried on until all patients were in absorption event (Death).

Note: Sampling from truncated Gompertz decrH.

Mathematically, a Gompertz distribution with monotonic decreasing hazard (call it, Gompertz decrH) has a non-zero probability for a patient to have *infinite* event time (patient can live forever) [4]. When Gompertz decrH is used in the presented model, the simulation was constrained such that all simulated patients have *finite* event time (i.e., no patient lives forever). The constraint was implemented by resampling the patient's event time until at least one of the possible competing times for the event from "event i" has finite event time. Thus, to say, sampling from "*truncated Gompertz decrH*" distribution.

The consequence of truncation is that simulated data from truncated Gompertz have higher hazard (shorter time to events, i.e., event time distribution is shifted to the left) compared to the actual hazard used to simulate the data. Thus, the hazard rate re-estimated from simulated data (outHR) is higher than the hazard rate used to obtain the simulated data (inHR). The magnitude of the difference between outHR and inHR depends on the strength of inHR. The lower inHR, the higher the number of patients with "infinite time" to be resampled, the higher the difference between outHR and inHR.

For the non-personalized strategy (patients treated with first-line chemotherapies), only 0.8% of patients had their event time from L1T to L2T and Death resampled after first having "infinite times" in both time to events (i.e., from L1T to L2T and from L1T to Death). However, for a personalized strategy (patients treated with first-line targeted therapies or immunotherapy), about 12 percent of patients had their event time from L1T to L2T and Death resampled after first having "infinite times" in both times to events (i.e., from L1T to L2T and L1T to Death).

To simulate treatment effect from a randomized clinical trials (RCT) for targeted therapy or immunotherapy compared to chemotherapy in the personalized strategy, a progression-free hazard for L1T to L2T and from L1T to Death (inHR) had to be calibrated. Calibration was done such that simulated results for outHR equal the progression-free hazard rate from the RCT (rctHR). The outHR was re-estimated by a coxPH model with cut-off time of 20 months from the start of first-line treatment to mimic a limited follow-up in RCT. For example, for the EGFR subgroup treated with first-line gefitinib, inHR of 0.38 was used to have an outHR of 0.43 which matches to the rctHR in a network meta-analysis [5]. Table A4 provides the rctHR and inHR for each molecular subgroup simulated in the personalized strategy. For the Immunotherapy treated subgroup, inHR was given under 23 percent long-term survivor fraction and 0 percent long-term survivor fraction as described in Sect. "Adjustment of the non-personalized strategy to simulate a personalized treatment strategy" of the main text.

A.2 Tables

Table A1. Distribution of baseline characteristics for patients with advanced (inoperable) non-squamous NSCLC diagnosed between 2008 and 2014

Variable	Non-squamous Cell		
	Systemic	BSC	All
Year (%)			
2008	82 (10.7)	211 (14.7)	293 (13.3)
2009	91 (12.0)	205 (14.3)	296 (13.5)
2010	95 (12.5)	208 (14.5)	303 (13.8)
2011	109 (14.3)	2015 (15.0)	324 (14.8)
2012	137 (18.0)	195 (13.6)	332 (15.1)
2013	126 (16.6)	192 (13.4)	318 (14.5)
2014	121 (16.0)	209 (14.6)	330 (15.0)
Gender (%)			
Male	432 (56.8)	849 (59.2)	1281 (58.3)
Female	329(43.2)	587 (40.8)	915 (41.7)
Age			
Mean ± std. Error	62.5 ± 0.350	68.9 ± 0.289	66.7 ± 0.234
PS (%)			
Good (0 – 1)	640 (84.1)	731 (50.9)	1371 (62.4)
Bad (2 – 4)	86 (11.3)	598 (41.7)	684 (31.1)
Unknown	35 (4.6)	106 (7.4)	141 (6.4)
CCI (%)			
Good (1 – 2)	718(94.3)	1266 (88.2)	1984 (90.3)
Bad (> 2)	43(5.7)	169 (11.8)	212 (9.7)
Histology (%)			
Adenocarcinoma	564 (74.1)	956 (66.6)	1520 (69.2)
Large cell	89 (11.7)	223 (15.5)	312 (14.2)
Other or NOS	108 (14.2)	256 (17.8)	364 (16.6)
First Line Treatment (%)			
Cisp. D			
Cisplatin-pemetrexed	291 (*38.2*)	-	291 (13.3)
Cisplatin-gemcitabine	90 (*11.8*)	-	90 (4.1)
Carbo. D			
Carboplatin-pemetrexed	195 (*25.6*)	-	195 (8.9)
Carboplatin-gemcitabine	87 (*11.4*)	-	87 (4.0)
Bevacizumab-carboplatin- paclitaxel	60 (*7.9*)	-	60 (2.7)
Carboplatin-docetaxel	38 (*5.0*)	-	38 (1.7)
BSC	-	-	65.34
Total	761 (34.7)	1435 (65.3)	2196 (100)

PS, Eastern Cooperative Oncology Group performance status; CCI, Charlson comorbidity index; Year, year of diagnosis; Age, age at diagnosis; BSC, best supportive care; Systemic, first-line systemic chemotherapy; Cisp. D, cisplatin doublet chemotherapy; Carbo. D, carboplatin doublet chemotherapy; std. Error – standard error of the mean.

NB. This table provides description of 2196 non-squamous NSCLC patients received first-line standard chemotherapy or best supportive care, for more details on the complete cohort, we refer to Cramer-van der Welle et al., 2018 [6].

Table A2. Bivariate association of baseline characteristics and first-line treatment decision for patients with advanced (inoperable) non-squamous NSCLC diagnosed between 2008 and 2014

Variables		Methods	Test-statistic and pvalues			% sim. pv <0.05
Variable 1	Variable 2		Statistic	df	pvalue	
Year	Gender	χ^2	1.94	6	0.925	6
	PS	χ^2	91.87	12	<0.0001	100
	CCI	χ^2	5.291	6	0.507	5
	Treat (Syst or BSC)	χ^2	19.82	6	0.003	97
	Syst (Cisp. D or Carb. D)	χ^2	8.01	6	0.237	7
	Age	Anova	1.971	6; 2189	0.064	6
Gender	PS	χ^2	2.97	2	0.227	25
	CCI	χ^2	14.34	1	0.0002	98
	Treat (Syst or BSC)	χ^2	1.078	1	0.299	44
	Syst (Cisp. D or Carb. D)	χ^2	4.07	1	0.044	8
	Age	ttest	6.10	1865.7	<0.0001	100
PS	CCI	χ^2	24.89	2	<0.0001	100
	Treat (Syst or BSC)	χ^2	240.87	2	<0.0001	100
	Syst (Cisp.D or Carb. D)	χ^2	10.61	2	0.005	93
	Age at Diagnosis	Anova	46.18	2; 2193	<0.0001	100
CCI	Treat (Syst or BSC)	χ^2	20.70	1	<0.0001	100
	Syst (Cisp. D or Carb. D)	χ^2	1.60	1	0.2059	100
	Age	ttest	-8.86	273.9	<0.0001	100
Treat (Syst or BSC)	Age	ttest	14.14	1727.7	<0.0001	100
Type of Syst (Cisp. D or Carb. D)	Age	ttest	-4.96	758.8	<0.0001	100

χ^2, Chi-square test of association; Anova, one way analysis of variance; ttest, two independent samples student's T test; PS, Eastern Cooperative Oncology Group performance status; CCI, Charlson comorbidity index; Year, year of diagnosis; Age, age at diagnosis; BSC, best supportive care; Syst, first-line systemic chemotherapy; Cisp. D, Cisplatin doublet chemotherapy; Carbo. D, Carboplatin doublet chemotherapy; % sim. pv ≤ 0.05, percentage of simulations with p-value less than 0.05 out of 1000 runs. Each run had 2196 simulated patients.

Table A3. Prevalence of molecular biomarkers in the population

Biomarker (subgroup)	Proportion	Standard error	Reference
$EGFR_{(classic)}$[a]	0.07	0.008	7
$EGFR_{(non-classic)}$[b]	0.01	0.001	7
ALK	0.02	0.002	7
ROS1	0.019	0.002	8
$BRAF_{(V600E)}$	0.021	0.002	9
$NTRK_{(1,2,3)}$	0.0017	-	10
$MET_{(amp\ /\ exon\ 14\ sk)}$	0.014	0.001	9
RET	0.017	0.002	9
KRAS	0.28	0.029	7
No / Unknown	0.5473[c]	-	-
For those tested for PD-L1			
PD-L1 ≥ 50%	0.25[d]		11
PD-L1 < 50%	0.75[e]	-	11

[a] Classic activating EGFR mutations (exon 19 deletions and exon 21 L858R point mutations).

[b] Non-classic activating EGFR mutations, resistance mutations, and other mutations.

[c] 1 minus sums all molecular markers.

[d] out of patients with unknown or without molecular biomarkers or had KRAS / MET / RET mutations.

[e] 1 minus PD-L1 ≥ 50%.

Table A4. First-line treatment and PFS HR per molecular subgroup

Biomarker	1L Treat	Control	PFS HR (95% CI)	Reference	inHR	
EGFR$_{(classic)}$[a]	Gefitinib	PDCT	0.43 (0.37 – 0.49)	[5]	0.3817	
EGFR$_{(classic)}$[a]	Erlotinib	PDCT	0.36 (0.30 – 0.43)	[5]	0.2518	
EGFR$_{(classic)}$	Prognostic value	-	0.82 (0.66 – 1.03)	[12] (dig)[c]	0.8009	
EGFR$_{(non\text{-}classic)}$ [b]	Afatinib	PDCT	0.40 (0.20 – 0.83)	[12c]	0.2586	
ALK	Alectinib	Crizotinib	0.47 (0.34 – 0.65)	[13]	-	
ALK	Crizotinib	PDCT	0.45 (0.35 – 0.60)	[14]	0.3339	
ALK	Alectinib	PDCT	0.212[d]	Assumption	0.0032[d]	
ALK	Prognostic value	-	0.80 (0.70 – 0.90)	[15]	0.7731	
ROS1	Crizotinib	-	0.45 (0.35 – 0.60)	[14,16 e]	0.3339	
BRAF$_{(V600E)}$	Dabrafenib & Trametinib	-	0.67 (0.53 – 0.84)	[17,18 f]	0.6247	
NTRK$_{(1,2,3)}$	Larotrectinib	-	0.82 (0.66 – 1.03)	[12,19 g]	0.8009	
MET$_{(amp\ /\ exon\ 14\ sk)}$	depends PD-L1	-	-	-	-	
RET	depends PD-L1	-	-	-	-	
HER2	depends PD-L1	-	-	-	-	
KRAS	depends PD-L1	-	-	-	-	
No / Unknown	depends PD-L1	-	-	-	-	
Long-term survivor fraction					23%	0%
PD-L1 ≥50%	Pembrolizumab	PDCT	0.50 (0.37 – 0.68)	[20]	0.7546	0.4070
PD-L1 ≥50%	Pembro & PDCT	PDCT	0.36 (0.25 – 0.52)	[21]	0.4376	0.1995
PD-L1 1-49%	Pembro & PDCT	PDCT	0.55 (0.34 – 0.90)	[21]	0.8715	0.4726

1L Treat, first-line treatment; Control, control arm treatment in randomized control trial (RCT); PFS HR, progression free hazard rate; CI, confidence interval; inHR, calibrated hazard rate used in Truncated Gompertz distribution to simulate cohort with novel treatment effect as the one observed in RCT (i.e., PFS HR).

[a] Classic activating EGFR mutations (exon 19 deletions and exon 21 L858R point mutations).

[b] Non-classic activating EGFR mutations, resistance mutations, and other mutations.

[c] Special request to the author to provide PFS HR, the method used can be obtained from the original work of Simons et al. [12].

[d] Assumed that the hazard ratio (HR) of alectinib compared to chemotherapy (PDCT) is equal to the product of HR of alectinib compared to crizotinib and HR of crizotonib compared to PDCT for patient with an ALK mutations i.e., 0.47*0.45 = 0.212.

[e] No literature evidence for PFS HR, it was assumed that treatment effect will be the same as crizotinib in ALK patients.

[f] No literature evidence for PFS HR, it was assumed that treatment effect will be the same as dabrafenib and trametinib in melanoma $BRAF_{val600}$ patients compared to dabrafenib and placebo.

[g] There is no evidence in the literature for PFS HR, we assumed that the effect of treatment will be the same as the prognostic value of $EGFR_{classic}$.

Table A5. Second-line treatment recommendation and PPFS HR per molecular subgroup and per first-line treatment

Biomarker	1L Treat	2L Treat	PPFS HR	Treat arm vs Control arm	References
EGFR (classic)	Gefitinib	Cis + Pem	N/A	-	-
EGFR (classic)	Erlotinib	Cis + Pem	N/A	-	-
EGFR (non-classic)	Afatinib	Cis + Pem	N/A	-	-
ALK	Alectinib	Lorlatinib	0.49 (0.37 – 0.64)	Crizotinib vs CT[a]	22
ROS1	Crizotinib	Cis + Pem	N/A	-	-
BRAF (V600E)	Dabraf & Tramet	PDL1 ≥ 50: Pemb	0.59 (0.44 – 0.78)	Pembro (2 gm/kg) vs CT	23
BRAF (V600E)	Dabraf & Tramet	PDL1 < 50: Pemb + Chem	0.59 (0.44 – 0.78)	Pembro (2 gm/kg) vs CT [b]	23
NTRK (1,2,3)	Larotrectinib	Cis + Pem	N/A	-	-
MET (amp / exon 14 sk)	depends PD-L1	-	-	-	-
RET	depends PD-L1	-	-	-	-
HER2	depends PD-L1	-	-	-	-
KRAS	depends PD-L1	-	-	-	-
No / Unknown	Depends PD-L1	-	-	-	-
PD-L1 ≥50%	Pembrolizumab	Cis + Pem	N/A	-	-
PD-L1 ≥50%	Pembro & PDCT	Docetaxel	N/A	-	-
PD-L1 1-49%	Pembro & PDCT	Docetaxel	N/A	-	-

1L Treat, first-line treatment; 2L Treat, second-line treatment; PPFS HR, post-progression free survival hazard ratio (i.e., Hazard ratio for 2L); CT, single dose chemotherapy (pemetrexed or docetaxel); Cis, Cisplatin, Pem, pemetrexed; Pembro, pembrolizumab; PDCT, platinum-based doublets; N/A, No adjustment on hazard rate.

We have assumed that, the treatment effect in second-line is independent of first-line treatment.

[a] Lorlatinib assumed to have similar effectiveness as second line crizotinib in ALK mutated patients.

[b] Assumed the effect of second line pembrolizumab monotherapy.

Table A6. Regression model describing the association between baseline characteristics and first-line treatment for patients with advance (inoperable) non-squamous NSCLC diagnosed between 2008 and 2014

Res. Var	Model type	Distribution	Scale	Covariate	Coefficient	95% CI
Age	Linear	normal	Linear	Intercept	67.88	67.28; 68.47
				Gender: F	-2.90	-3.83; -1.98
				Sigma	10.85	
CCI						
CCI (bad)	Logistic	Binomial	Logit	Intercept	-5.88	-6.98; -4.83
				Age	0.06	0.04; 0.07
				Gender: F	-0.49	-0.81; -0.18
PS						
PS (obs)	Logistic	Binomial	Logit	Intercept	1.84	1.52; 2.19
				Yr2009	1.16	0.55; 1.82
				Yr2010	0.19	-0.29; 0.68
				Yr2011	1.11	0.53; 1.74
				Yr2012	0.46	-0.03; 0.97
				Yr2013	2.52	1.59; 3.73
				Yr2014	3.26	2.06; 5.08
PS (bad)[a]	Logistic	Binomial	Logit	Intercept	-3.32	-3.99; -2.66
				Yr2009	-0.01	-0.37; 0.36
				Yr2010	0.16	-0.21; 0.52
				Yr2011	-0.04	-0.39; 0.32
				Yr2012	-0.49	-0.86; -0.12
				Yr2013	-0.21	-0.57; 0.15
				Yr2014	-0.27	-0.63; 0.08
				Age	0.04	0.03; 0.05
				CCI: bad	0.52	0.22; 0.83
Treat						
Treat (sys)	Logistic	Binomial	Logit	Intercept	2.78	2.12; 3.44
				Yr2009	0.03	-0.36; 0.42
				Yr2010	0.27	-0.12; 0.65
				Yr2011	0.29	-0.08; 0.67
				Yr2012	0.57	0.21; 0.94
				Yr2013	0.48	0.11; 0.85
				Yr2014	0.41	0.04; 0.78
				Age	-0.05	-0.06; -0.04
				PS: bad	-1.64	-1.90; -1.39
				PS: miss	-0.81	-1.24; -0.40
				CCI: bad	-0.39	-0.77; -0.01
Type of Sys[b]						
Sys (Carbo)	Logistic	Binomial	Logit	Intercept	-2.51	-3.52; -1.53
				PS: bad	0.58	0.11; 1.06
				PS: miss	-0.87	-1.65; -0.15
				Age	0.04	0.02; 0.06

Res. Var, response variable; Model type, regression model type; Distribution, type of statistical distribution assumed for Res. Var; Scale, scale of linear predictor used to model Res. Var via Model type (e.g., if Res. Var assumed to have a normal distribution, then linear scale is used to model via linear regression); 95% CI, 95% confidence interval of regression coefficients (lower; upper); Age, age at diagnosis; F, Female; sigma, standard error of linear regression; CCI,

Charlson comorbidity Index, grouped as good or bad; PS, Eastern Cooperative Oncology Group Performance Status (obs, PS was observed and grouped as bad or good); Yr, year of diagnosis; Treat, decision to treat with systemic chemotherapy (Sys) or best supportive care (BSC); Type of Sys,Type of systemic thermotherapy administered, grouped as Carboplatin doublets (Carbo. D) and Cisplatin doublets (Cisp.D).

[a] fitted on a subset of patients with observed PS.

[b] fitted on a subset of patients received systemic chemotherapy.

Table A7. Time-to-event estimates of disease model (Fig. 1)

Term	Trans01[a]	Trans04[a]	Trans12[b]	Trans14[b]	Trans23[b]	Trans24[b]	Trans34[b]
Distribution	*Log-logistic*	*Log-normal*	*Gompertz*	*Gompertz*	*Exponential*	*Exponential*	*Exponential*
Shape	0.9970*	-0.2110*	-0.2993^{+}	-0.3369^{+}	-	-	-
Intercept	-2.5490*	-1.3330*	0.5231^{+}	0.1003^{+}	-1.3590	-0.252	-0.363
Treat1g: Carb. D	-	-	0.1969	0.1457	0.4420	0.501*	0.005
PS:	-	-	-	-	-	-	-
Bad	-	0.8150*	-	0.4295*	-	-	-
Unkn	-	0.8250*	-	0.2050	-	-	-
Gender: Female	-0.107*	-0.1340*	-0.3281*	-	-0.814*	-0.510*	-
Age	-	-	-0.0233*	-	-0.044*	-	-

Treat1g, first-line systemic treatment groups; Carb. D, Carboplatin doublets compared to Cisplatin doublets; PS, ECOG performance status (Bad, ie., PS 2 or higher, Unkn, ie., unknown PS compared to good, ie., PS 0 or 1); Age, age at diagnosis in years.

Events: 0 is DIAG, diagnosis of advanced (inoperable) NSCLC; 1 is L1T, start of first-line treatment; 2 is L2T, start of second-line treatment; 3 is L3T, the start of third-line treatment; and 4 is death.

Time-to-events: Trans01, from DIAG to L1T; Trans04, from DIAG to death; Trans12, from L1T to L2T; Trans14, from L1T to death; Trans23, from L2T to L3T; Trans24, from L2T to death; Trans34, from L3T to death. (see Fig. 1, of the main text).

* Significant at alpha level of 0.05.

$^{+}$ p-value / CI not reported.

[a] Fitted as sub-distribution model.

[b] fitted as parametric multistate statistical model (parMSSM).

Table A8. Comparison of real-world, RCT, and modelled survival estimates

	Real-world			RCT	Modelled	
	Netherlands	US				
	Cramer-van der Welle et al., [24]	Velcheti et al., [25 a]	Waterhouse et al., [26 b]		Cure rate: 0%	Cure rate: 23%
**PD-L1 ≥ 50%; Pembrolizumab monotherapy**				5 year update Keynote-024 [27]		
mPFS	8.9 (3.7–14.1)	NA	NA	7.7 (6.1-10.2)	11.5	10.9
mOS	15.8 (9.4–22.1)	NA	15.3 (13.4-17.5)	26.3 (18.3-40.4)	13.6	13.1
Surv. Rate (%)						
12 m	57	NA	54.5	NA	53.8	52.3
24 m	NA	NA	39.6	NA	29.6	35.9
36 m	NA	NA	31.6	43.7	16.7	28.2
**PD-L1 ≥ 50; Pembrolizumab + PDCT**				3 year update keynote-189 [28]		
mPFS	NA	8.8 (7.2-11.6)	NA	11.1 (9.1-14.4)	16.6	17.1
mOS	NA	20.6 (14.8-NR)	19.1 (15.5-22.1)	NR (20.4-NR)	18.9	19.3
Surv. Rate (%)						
6 m	NA	80.7 (70.1-87.9)	NA		80.5	78.4
12 m	NA	65.1 (53.3-74.5)	61.0	73.3	64.6	62.9
18 m	NA	54.6 (42.2-65.5)	NA	NA	51.7	52.1
24 m	NA	NA	42.7	51.9	41.1	44.1
**PD-L1 1-49%; Pembrolizumab + PDCT**						
mPFS	NA	5.9 (4.6-8.1)	NA	9.2 (7.8-13.1)	10.4	9.3
mOS	NA	16.3 (11.6-22.4)	11.8 (10.5-13.5)	21.8 (17.7-25.9)	12.3	11.5
Surv. Rate (%)						
6 m	NA	76.0 (64.6-84.1)	NA	NA	71.6	67.0
12 m	NA	59.6 (47.5-69.8)	49.1	71.7	50.6	48.9
18 m	NA	47.5 (35.6-58.6)	NA	NA	36.9	38.7
24 m	NA	NA	29.5	44.3	27.2	32.6

PDCT, platinum-based doublet chemotherapy; CT, pemetrexed; Surv. rate, overall survival rate; mPFS, median progression-free survival time; mOS, median overall survival time; m, months; NA, not available; NR, not reached; US, United States of America; RCT, randomized control trial.

[a] Contains patients with only good performance status i.e., ECOG PS 0 - 1;

[b] The Study contains different types of immunotherapies, for immunotherapy monotherapy, 95% of patients had pembrolizumab, and for combination of immunotherapy with chemotherapy, 97% of patients had pembrolizumab plus PDCT/CT;

A.3 Figures

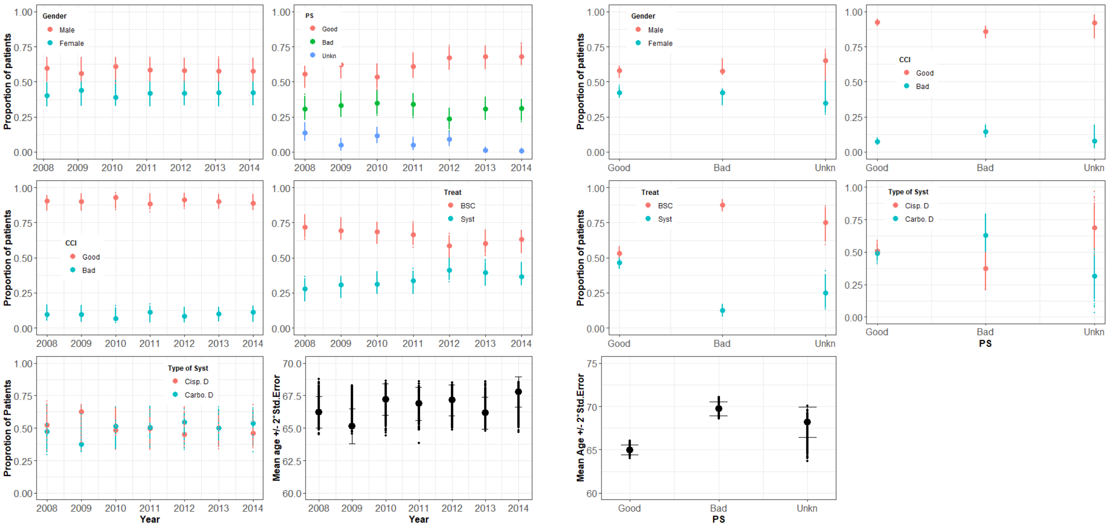

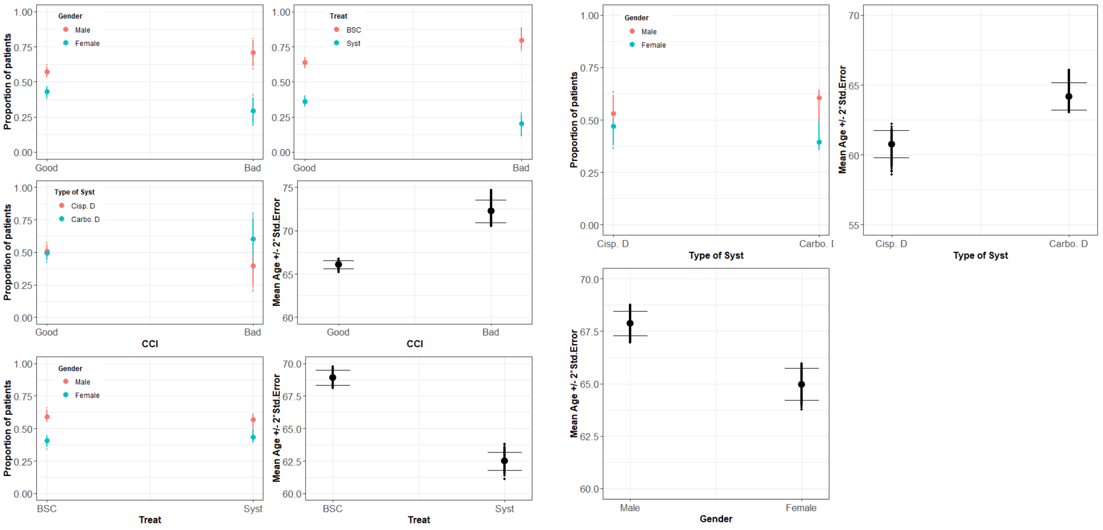

Figure A1. Comparison of modelled and observed bivariate association of baseline attributes and treatment for advanced (inoperable) NSCLC.

PS, Eastern Cooperative Oncology Group performance status; CCI, Charlson comorbidity index; BSC, best supportive care; Syst, systemic chemotherapy; Cisp. D, Cisplatin doublet chemotherapy; Carbo. D, Carboplatin doublet chemotherapy.

N.B., Statistical test results are reported in ESM Table 2 on column “% sim. pv <0.05”.

Large dots indicate the observed values (with 95% confidence interval from the template dataset).

Small dots indicate simulated values from 1000 runs of a non-personalized strategy, each run had 2196 simulated patients (sample size of template data).

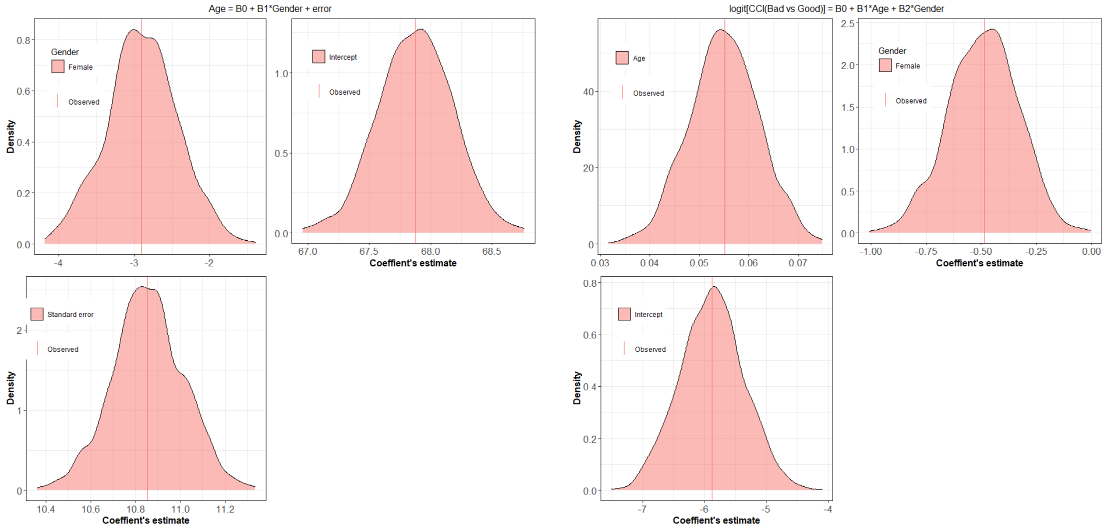

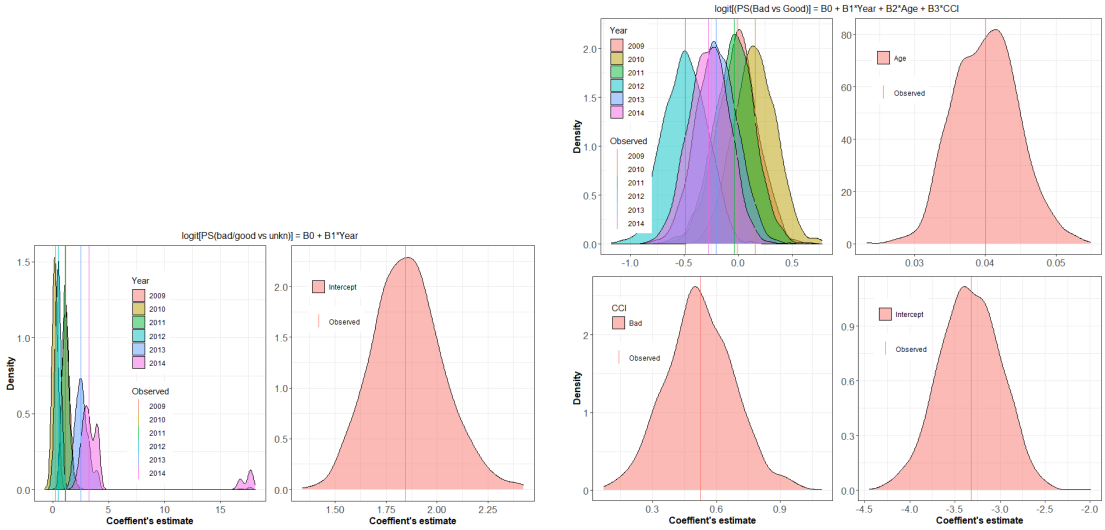

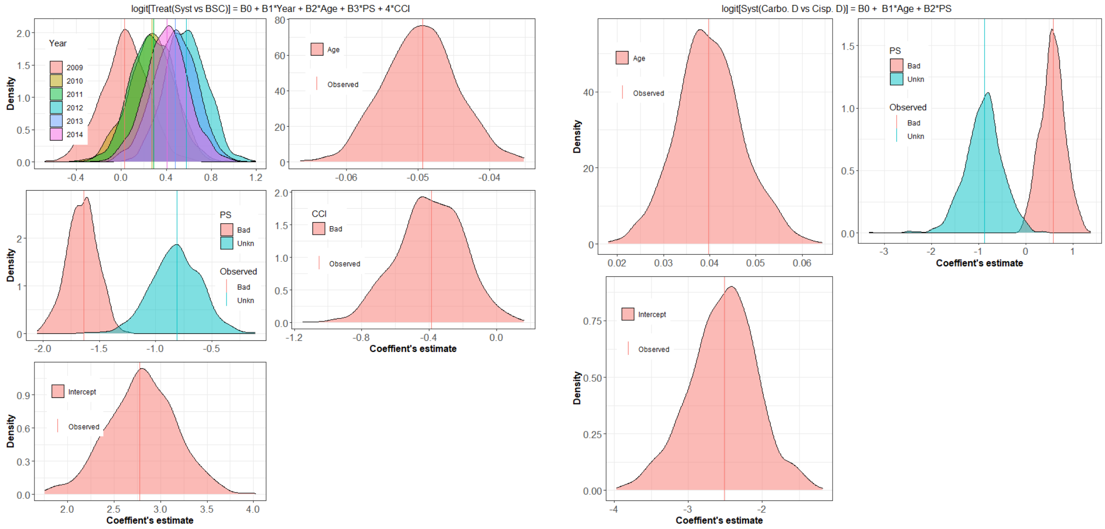

Figure A2. Distribution of parameters of regression models used to simulate baseline attributes and treatment decision of base-case model (a non-personalized strategy).

The model equations are given on top of each panel.
B0, intercept; error, random error with standard normal distribution; PS, Eastern Cooperative Oncology Group performance status; CCI, Charlson comorbidity index; Year, year of diagnosis; Age, age at diagnosis; BSC, best supportive care; Syst, systemic chemotherapy; Cisp. D, Cisplatin doublet chemotherapy; Carbo. D, Carboplatin doublet chemotherapy; Observed, parameter values obtained by fitting regression model on template dataset.

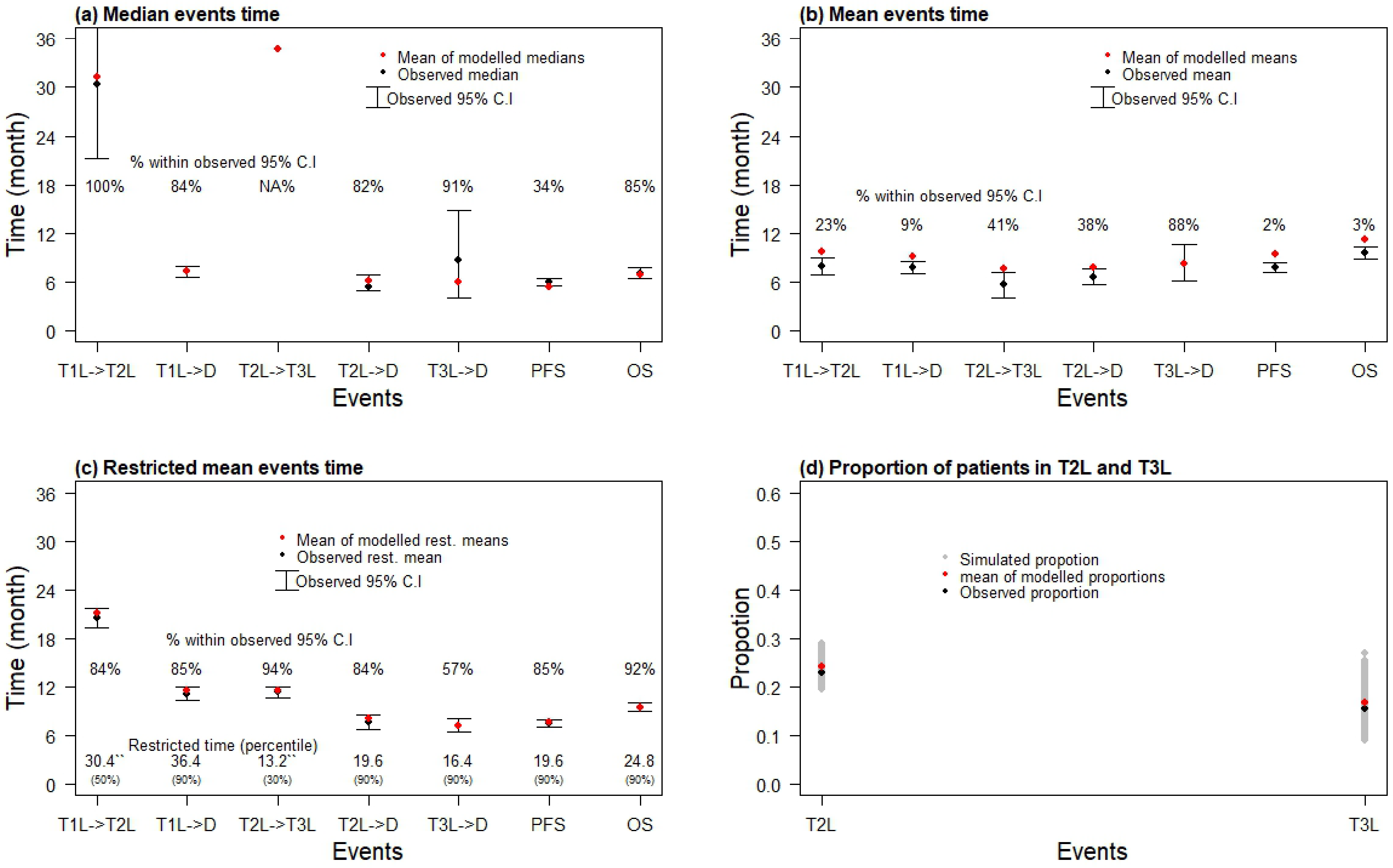
(a) Median events time
Mean of modelled medians
Observed median
Observed 95% C.I
% within observed 95% C.I
100% 84% NA% 82% 91% 34% 85%
Time (month)
0 6 12 18 24 30 36
T1L->T2L T1L->D T2L->T3L T2L->D T3L->D PFS OS
Events
(b) Mean events time
Mean of modelled means
Observed mean
Observed 95% C.I
% within observed 95% C.I
23% 9% 41% 38% 88% 2% 3%
Time (month)
0 6 12 18 24 30 36
T1L->T2L T1L->D T2L->T3L T2L->D T3L->D PFS OS
Events
(c) Restricted mean events time
Mean of modelled rest. means
Observed rest. mean
Observed 95% C.I
% within observed 95% C.I
84% 85% 94% 84% 57% 85% 92%
Restricted time (percentile)
30.4`` 36.4 13.2`` 19.6 16.4 19.6 24.8
(50%) (90%) (30%) (90%) (90%) (90%) (90%)
Time (month)
0 6 12 18 24 30 36
T1L->T2L T1L->D T2L->T3L T2L->D T3L->D PFS OS
Events
(d) Proportion of patients in T2L and T3L
Simulated propotion
mean of modelled proportions
Observed proportion
Propotion
0.0 0.1 0.2 0.3 0.4 0.5 0.6
T2L T3L
Events

Figure A3. Comparison of modelled and observed time to event and proportion of events.

The modelled median, mean, restricted (rest.) mean and proportion in L2T and L3T were evaluated from 1000 runs of a non-personalized strategy.

Observed median, mean, and restricted mean were computed from the template dataset (Santeon registry 2008-2014).

L1T, L2T, L3T, and D, are first-line, second-line, third-line treatment, and death events, respectively.

PSF, progression-free survival; OS, overall survival; C.I, confidence interval; NA, not reached.

i->j, from the *i event* to the *j event.* For *i and j* = L1T, L2T, L3T, and D.

`` For panel c, restricted time was taken at the maximum percentile estimable ie., 50th percentile for T1L->T2L and 30th percentile for T2L->T3L.

All remaining events, the restricted time was taken at the 90th percentile.

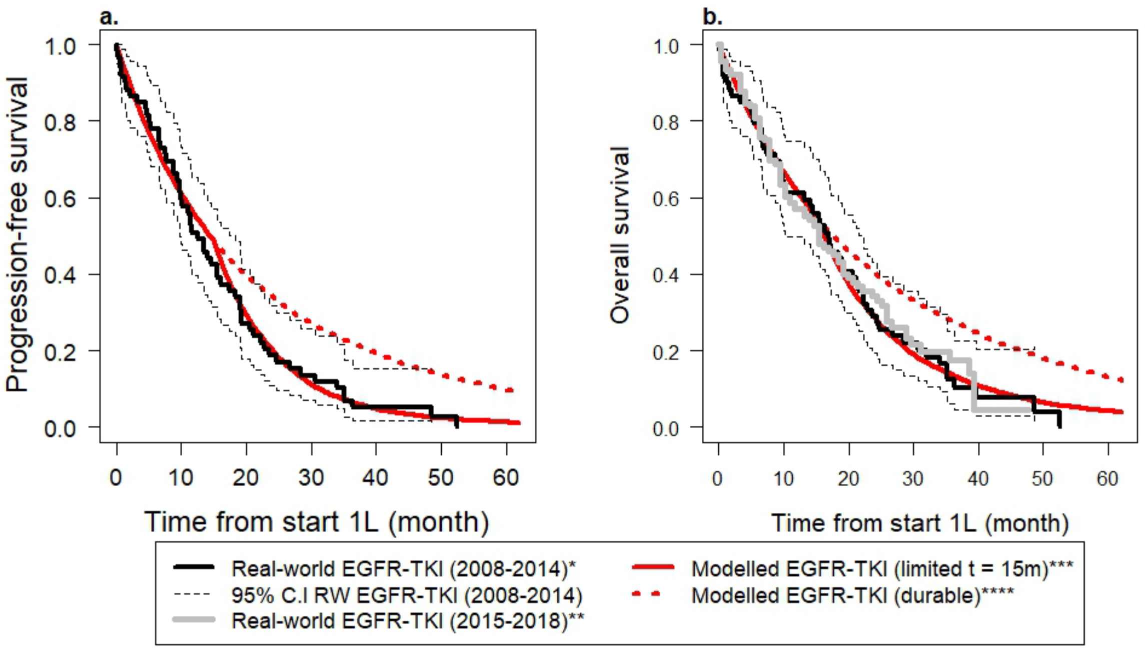

Figure A4. Comparison of modelled and real-world progression-free survival (a) and overall survival (b) curves for patients with epidermal growth factor receptor (EGFR) mutations and treated with the fist-line EGFR tyrosine kinase inhibitors (EGFR-TKIs).

EGFR-TKIs were gefitinib or erlotinib; C.I, confidence interval; RW, Real-world; 1L, start of first-line systemic treatment;

* The real-world data of 52 patients diagnosed and treated between 2008 and 2014 in Santeon hospitals [29].

** The reconstructed (digitized) real-world overall survival (OS) data of 147 patients treated between 2015 and 2018 in Santeon hospitals. The OS data were digitized from the curve published by Cramer-van der Welle et al., 2021 [24]. Progression-free survival (PFS) data were not available.

*** Modelled data was simulated assuming EGFR-TKI benefit wear out after 15 months (t = 15m).

**** Modelled was simulated assuming a durable EGFR-TKIs benefit i.e., benefit last until disease progression or death (durable).

NB: The hazard ratios of EGFR-TKI were 0.43 and 0.36 for gefitinib and erlotinib compared to chemotherapy, respectively [5], and hazard ratio of 0.82 for prognostic value of EGFR positive compared to EGFR negative [12].

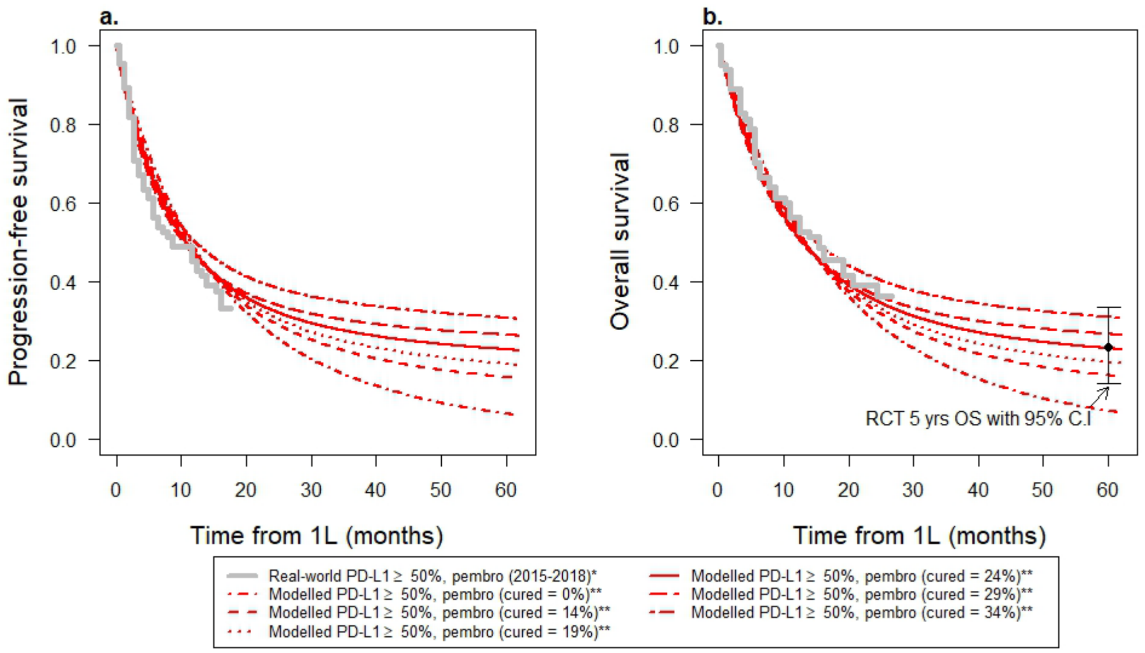

Figure A5. Results of a deterministic sensitivity analysis on the impact of the long-term survivor fraction (cure rate) on progression-free survival (a) and overall survival (b) for patients with a high programmed death ligand 1 (PD-L1 ≥ 50%) expression and treated with a first-line pembrolizumab monotherapy.

Cured, long-term survivor fraction.

* The reconstructed (digitized) progression-free and overall survival data of 83 patients diagnosed and treated between 2015 and 2018 in Santeon hospitals [24].

** Modelled data was simulated assuming different values of a long-term survivor fraction (cured) and a hazard ratio of 0.5 for pembrolizumab compared to chemotherapy [20].

The black dot and interval bar (RCT 5-yr OS with 95% C.I) indicates a five-year overall survival with 95% confidence interval from Keynote-001 [30].

3 REFERENCES

1 Putter, H., Fiocco, M. & Geskus, R. B. Tutorial in biostatistics: competing risks and multi-state models. *Stat Med* **26**, 2389-2430, doi:10.1002/sim.2712 (2007).

2 Beyersmann, J., Allignol, A. & Schumacher, M. *Competing Risks and Multistate Models with R*. (Springer, 2012).

3 Williams, C., Lewsey, J. D., Briggs, A. H. & Mackay, D. F. Cost-effectiveness Analysis in R Using a Multi-state Modeling Survival Analysis Framework: A Tutorial. *Med Decis Making* **37**, 340-352, doi:10.1177/0272989X16651869 (2017).

4 Package ' flexsurv' (CRAN, 2019).

5 Holleman, M. S., van Tinteren, H., Groen, H. J., Al, M. J. & Uyl-de Groot, C. A. First-line tyrosine kinase inhibitors in EGFR mutation-positive non-small-cell lung cancer: a network meta-analysis. *Onco Targets Ther* **12**, 1413-1421, doi:10.2147/OTT.S189438 (2019).

6 Cramer-van der Welle, C. M. *et al.* Systematic evaluation of the efficacy-effectiveness gap of systemic treatments in metastatic nonsmall cell lung cancer. *Eur Respir J* **52**, doi:10.1183/13993003.01100-2018 (2018).

7 Kuijpers, C. *et al.* Association of molecular status and metastatic organs at diagnosis in patients with stage IV non-squamous non-small cell lung cancer. *Lung Cancer* **121**, 76-81, doi:10.1016/j.lungcan.2018.05.006 (2018).

8 Skoulidis, F. & Heymach, J. V. Co-occurring genomic alterations in non-small-cell lung cancer biology and therapy. *Nat Rev Cancer* **19**, 495-509, doi:10.1038/s41568-019-0179-8 (2019).

9 Jordan, E. J. *et al.* Prospective Comprehensive Molecular Characterization of Lung Adenocarcinomas for Efficient Patient Matching to Approved and Emerging Therapies. *Cancer Discov* **7**, 596-609, doi:10.1158/2159-8290.CD-16-1337 (2017).

10 Forsythe, A. *et al.* A systematic review and meta-analysis of neurotrophic tyrosine receptor kinase gene fusion frequencies in solid tumors. *Ther Adv Med Oncol* **12**, 1758835920975613, doi:10.1177/1758835920975613 (2020).

11 Dietel, M. *et al.* Real-world prevalence of programmed death ligand 1 expression in locally advanced or metastatic non-small-cell lung cancer: The global, multicenter EXPRESS study. *Lung Cancer* **134**, 174-179, doi:10.1016/j.lungcan.2019.06.012 (2019).

12 Simons, M. *et al.* Observed versus modelled lifetime overall survival of targeted therapies and immunotherapies for advanced non-small cell lung cancer patients - A systematic review. *Crit Rev Oncol Hematol* **153**, 103035, doi:10.1016/j.critrevonc.2020.103035 (2020).

13 Peters, S. *et al.* Alectinib versus Crizotinib in Untreated ALK-Positive Non-Small-Cell Lung Cancer. *N Engl J Med* **377**, 829-838, doi:10.1056/NEJMoa1704795 (2017).

14 Solomon, B. J. *et al.* First-line crizotinib versus chemotherapy in ALK-positive lung cancer. *N Engl J Med* **371**, 2167-2177, doi:10.1056/NEJMoa1408440 (2014).

15 Wang, Z. L. *et al.* Anaplastic lymphoma kinase gene rearrangement predicts better prognosis in NSCLC patients: A meta-analysis. *Lung Cancer* **112**, 1-9, doi:10.1016/j.lungcan.2017.07.029 (2017).

16 Shaw, A. T. *et al.* Crizotinib in ROS1-rearranged advanced non-small-cell lung cancer (NSCLC): updated results, including overall survival, from PROFILE 1001. *Ann Oncol* **30**, 1121-1126, doi:10.1093/annonc/mdz131 (2019).

17 Long, G. V. *et al.* Dabrafenib and trametinib versus dabrafenib and placebo for Val600 BRAF-mutant melanoma: a multicentre, double-blind, phase 3 randomised controlled trial. *Lancet* **386**, 444-451, doi:10.1016/S0140-6736(15)60898-4 (2015).

18 Kelly, R. J. Dabrafenib and trametinib for the treatment of non-small cell lung cancer. *Expert Rev Anticancer Ther* **18**, 1063-1068, doi:10.1080/14737140.2018.1521272 (2018).

19 Hong, D. S. *et al.* Larotrectinib in patients with TRK fusion-positive solid tumours: a pooled analysis of three phase 1/2 clinical trials. *Lancet Oncol* **21**, 531-540, doi:10.1016/S1470-2045(19)30856-3 (2020).

20 Reck, M. *et al.* Pembrolizumab versus Chemotherapy for PD-L1-Positive Non-Small-Cell Lung Cancer. *N Engl J Med* **375**, 1823-1833, doi:10.1056/NEJMoa1606774 (2016).

21 Gandhi, L. *et al.* Pembrolizumab plus Chemotherapy in Metastatic Non-Small-Cell Lung Cancer. *N Engl J Med* **378**, 2078-2092, doi:10.1056/NEJMoa1801005 (2018).

22 Shaw, A. T. *et al.* Crizotinib versus chemotherapy in advanced ALK-positive lung cancer. *N Engl J Med* **368**, 2385-2394, doi:10.1056/NEJMoa1214886 (2013).

23 Herbst, R. S. *et al.* Pembrolizumab versus docetaxel for previously treated, PD-L1-positive, advanced non-small-cell lung cancer (KEYNOTE-010): a randomised controlled trial. *Lancet* **387**, 1540-1550, doi:10.1016/S0140-6736(15)01281-7 (2016).

24 Cramer-van der Welle, C. M. *et al.* Real-world outcomes versus clinical trial results of immunotherapy in stage IV non-small cell lung cancer (NSCLC) in the Netherlands. *Sci Rep* **11**, 6306, doi:10.1038/s41598-021-85696-3 (2021).

25 Velcheti, V., Hu, X. H., Piperdi, B. & Burke, T. Real-world outcomes of first-line pembrolizumab plus pemetrexed-carboplatin for metastatic nonsquamous NSCLC at US oncology practices. *Sci Rep-Uk* **11**, doi:ARTN 922210.1038/s41598-021-88453-8 (2021).

26 Waterhouse, D. *et al.* Real-world outcomes of immunotherapy-based regimens in first-line advanced non-small cell lung cancer. *Lung Cancer* **156**, 41-49, doi:10.1016/j.lungcan.2021.04.007 (2021).

27 Reck, M. *et al.* Five-Year Outcomes With Pembrolizumab Versus Chemotherapy for Metastatic Non-Small-Cell Lung Cancer With PD-L1 Tumor Proportion Score >/= 50. *J Clin Oncol* **39**, 2339-2349, doi:10.1200/JCO.21.00174 (2021).

28 Gadgeel, S. *et al.* Updated Analysis From KEYNOTE-189: Pembrolizumab or Placebo Plus Pemetrexed and Platinum for Previously Untreated Metastatic Nonsquamous Non-Small-Cell Lung Cancer. *J Clin Oncol* **38**, 1505-1517, doi:10.1200/JCO.19.03136 (2020).

29 Sluga, R. *et al.* Utilization of Molecular Testing and Survival Outcomes of Treatment with First- or Second-line Tyrosine Kinase Inhibitors in Advanced Non-small Cell Lung Cancer in a Dutch Population. *Anticancer Res* **38**, 393-400, doi:10.21873/anticanres.12235 (2018).

30 Garon, E. B. *et al.* Five-Year Overall Survival for Patients With Advanced NonSmall-Cell Lung Cancer Treated With Pembrolizumab: Results From the Phase I KEYNOTE-001 Study. *J Clin Oncol* **37**, 2518-2527, doi:10.1200/JCO.19.00934 (2019).
